# Role of Spacers in Molecularly Linked RuRh Dyads: A Comparative Synthetic and Ultrafast Spectroscopic Investigation

**DOI:** 10.1021/acs.inorgchem.4c04596

**Published:** 2025-04-10

**Authors:** Mohini Semwal, Martin Lämmle, Elias H. P. Brohmer, Steffen Volk, Linda Zedler, Stephan Kupfer, Alexander K. Mengele, Georgina E. Shillito, Sven Rau, Benjamin Dietzek-Ivanšić

**Affiliations:** a Institute of Physical Chemistry, Friedrich Schiller University Jena, Helmholtzweg 4, Jena 07743, Germany; b Research Department Functional Interfaces, Leibniz Institute of Photonic Technology, Albert-Einstein-Str. 9, Jena 07745, Germany; c Institute of Inorganic Chemistry I, Ulm University, Albert-Einstein-Allee 11, Ulm 89081, Germany; d Zentrum für Sonnenenergie- und Wasserstoff-Forschung Baden-Württemberg, Helmholtzstraße 8, Ulm 89081, Germany; e Institute of Organic Chemistry I, Ulm University, Albert-Einstein-Allee 11, Ulm 89081, Germany; f Center for Energy and Environmental Chemistry Jena (CEEC Jena), Friedrich Schiller University Jena, Lessingstraße 8, Jena 07743, Germany

## Abstract

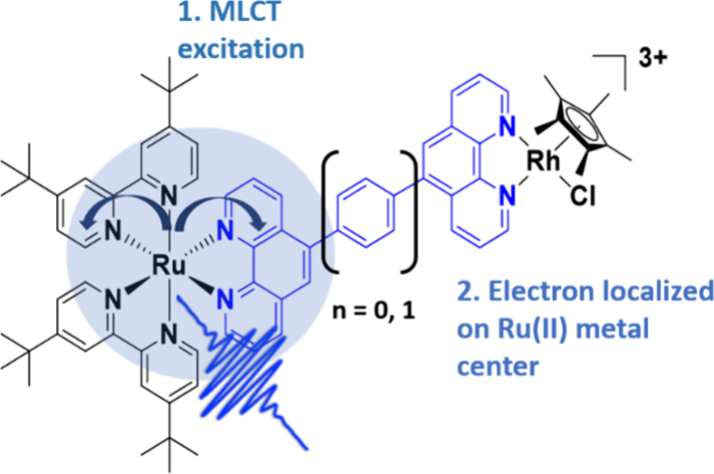

Supramolecular photocatalysts consisting of photosensitizer (PS), bridging ligand (BL), and catalytic center (CAT) have garnered significant attention in solar fuel applications. In this study, the photophysics and photocatalytic properties of two Ru(II)-based dinuclear complexes, specifically [(tbbpy)_2_Ru(p(Ph)_*n*_p)Rh(Cp*)Cl]^3+^ (*n* = 0, 1; **Ru(pp)Rh** for *n* = 0 or **Ru(p(Ph)p)Rh** for *n* = 1; tbbpy = 4,4′-di-*tert*-butyl-2,2′-bipyridine, Cp* = pentamethylcyclopentadienyl, Ph = phenyl, p = 1,10-phenanthroline), are investigated. These complexes are studied as model complexes only differing by the distance between PS and CAT and thus allows a selective investigation of the influence of spacers in light-driven catalysis. A joint synthetic, spectroscopic, and theoretical approach, incorporating time-resolved absorption and emission spectroscopy, resonance Raman (rR) spectroscopy, density functional theory (DFT), and time-dependent (TD)DFT calculations, allows for comprehensive structural, electrochemical, photophysical, and photochemical characterization. Our findings suggest that minor structural variations in the intramolecular photocatalytic system significantly impact photocatalytic activity and system stability.

## Abstract 2

Ru(II)-based dinuclear complexes, specifically [(tbbpy)_2_Ru(p(Ph)_*n*_p)Rh(Cp*)Cl]^3+^ (*n* = 0, 1; **Ru(pp)Rh** for *n* = 0 or **Ru(p(Ph)p)Rh** for *n* = 1; tbbpy = 4,4′-di-*tert*-butyl-2,2′-bipyridine, Cp* = pentamethylcyclopentadienyl, Ph = phenyl, p = 1,10-phenanthroline), were investigated in solution. The light-driven electronic processes are investigated through a combination of ultrafast spectroscopy and quantum chemical calculations. The choice of a spacer determines the photophysical and photochemical properties and consequently the resulting photocatalytic activity.

## Introduction

Supramolecular photocatalysis, especially within the framework of artificial photosynthesis, presents a promising approach to address critical challenges such as climate change and the growing global demand for energy.^1,2^ A key area of interest within supramolecular systems is the class of covalently linked donor–acceptor systems, which have garnered significant attention in photochemistry.^3^ Among them, the simplest two-component ″dyads″ provide an excellent platform for investigating photoinduced electron and energy transfer mechanisms. Indeed, studies on dyads have greatly enhanced our understanding of how essential physical factors—such as energy gradients, the distance between components, intervening bonds, and the surrounding medium—affect the kinetics of electron and energy transfer.^4^

Our team has made pioneering contributions to the photocatalysis and photophysics of a class of supramolecular photocatalysts based on the lead structure [(tbbpy)_2_Ru(tpphz)MX_2_]^2+^ (CAT with M = Pd, Pt; X = Cl in the case of Pd and X = Cl or I in the case of Pt; tpphz = tetrapyrido[3,2-a:2′,3′-c:3″,2″-h:2‴,3‴-j]phenazine). In these systems, the octahedrally N-surrounded Ru(II) fragment constitutes the visible light absorbing PS, which is linked to the CAT by a tpphz BL. However, it was shown that the Pd-containing system readily undergoes degradation during catalysis, while the Pt system is stable.^5,6^ The instability of these dinuclear systems was also shown for other systems, e.g., [(tbbpy)_2_Ru(pp)PtCl_2_]^2+^ (tbbpy = 4,4′-di-*tert*-butyl-2,2′-bipyridine, pp = 5,5′-bis-1,10-phenanthroline), indicating a crucial effect of the BL constitution on the mechanism and output during light-driven catalysis.^7^ To overcome this often-occurring side reaction of metal particle formation, replacement of an unstable CAT with a more robust one is desired. In this regard, the introduction of a RhCp* (Cp* = pentamethylcyclopentadienyl) moiety as a CAT presents an attractive solution. These RhCp* complexes are known for their chemical stability under various conditions, making thus-derived supramolecular catalysts suitable candidates for long-term catalytic applications. Furthermore, the Rh(III) center can be electrochemically and photochemically reduced by one or two electrons resulting in distinct changes to its properties, allowing more detailed mechanistic insights.^8^ This strategy represents a promising avenue for advancing the development of efficient and durable photocatalytic systems for solar fuel production.^9^

**Figure 1:**
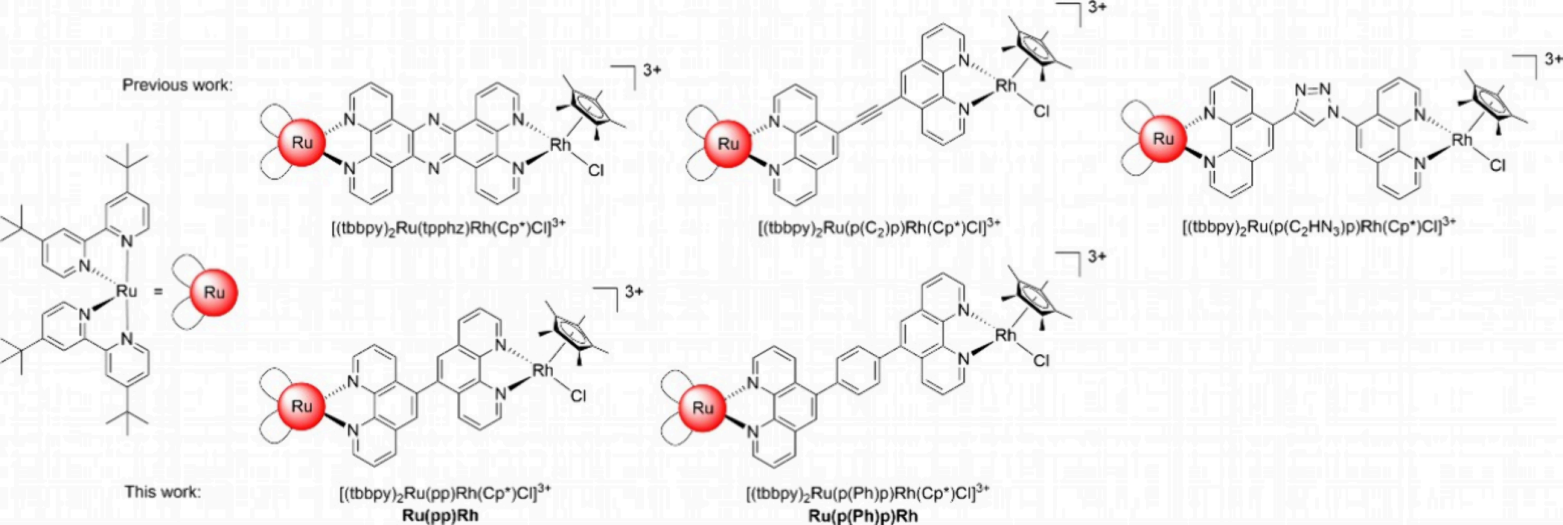
Molecular structures of selected examples of previously published dinuclear RuRh-based NAD^+^-reducing photocatalysts (top, p(C_2_)*p* = 1,2-di(1,10-phenanthrolin-5-yl)ethyne)) and the newly synthesized complexes (bottom **Ru(pp)Rh** and **Ru(p(Ph)p)Rh**).^16^ All complexes contain one Cl^–^ and two PF_6_^–^ counterions.

In the context of the influence of BLs on intramolecular electron transfer (eT), previous studies have highlighted that BLs can provide a conjugated system that allows ultrafast long-range charge separation between PS and CAT.^5,10−15^ Our group previously pointed out that conjugated systems promote eT to the CAT through studies of structurally related systems (see Figure 1, top row).^16^ Nevertheless, the triazole-spaced BL, which was also a conjugated system but behaved as an insulator to the system p(C_2_HN_3_)p (5,5′-(1*H*-1,2,3-triazole-1,4-diyl)bis(1,10-phenanthroline)), see Figure 1), still allowed light-driven NAD^+^ (nicotinamide adenine dinucleotide) reduction in a RuRh dyad, i.e., oxidative quenching of PS by CAT is not necessary to induce photocatalysis.^16^ Tamaki and Ishitani also reported superior performance by a nonconjugated C_2_H_4_-spaced bis-2,2′-bipyridine bridging ligand supramolecular photocatalyst (RuRe) for CO_2_ reduction, but this was explained by an optimized redox potential at the CAT.^17^ In the same line, Sauvage and co-workers highlighted the role of nonconjugated spacer units in heterometallic RuRh dyads with (tpy(Ph)_*n*_tpy) (*n* = 0, 1, 2; tpy = 2,2′:6′,2″-terpyridine) BLs where eT only takes place in the system of shortest bridging distance of Ru to Rh (*n* = 0).^4,18^ Although eT rates commonly decrease with increasing BL length due to a twist-induced diminished electronic coupling,^19−21^ systematic understanding of the nature of the BL in intramolecular photocatalytic systems is needed to understand this effect in multielectron photocatalysis.^12^

Based on this ground, two new supramolecular photocatalysts [(tbbpy)_2_Ru(p(Ph)_*n*_p)Rh(Cp*)Cl]Cl(PF_6_)_2_ (*n* = 0, 1; **Ru(pp)Rh** for *n* = 0 or **Ru(p(Ph)p)Rh** for *n* = 1) only differing in BL length by introduction of one additional phenyl group (*n* = 1: BL is p(Ph)p = 1,4-di(1,10-phenanthrolin-5-yl)benzene) are explored. We discuss the synthesis, electronic structure, photophysical properties, and catalytic performance of both RuRh bimetallic complexes. Through a combination of experimental techniques and quantum chemical studies, insight into the electronic states, eT processes, and light-driven reactivity are obtained. In particular, the electronic excited states are probed by resonance Raman (rR) spectroscopy at different excitation wavelengths, while the photoinduced reaction pathways are investigated by pump-wavelength and solvent-dependent time-resolved transient absorption (TA) spectroscopy. The excitation wavelength-dependent TA studies are key to elucidating the excited-state pathways. Finally, the long-lived emissive state is investigated through nanosecond transient emission spectroscopy.

## Results and Discussion

### Synthesis and Structural Characterization

**Scheme 1:**
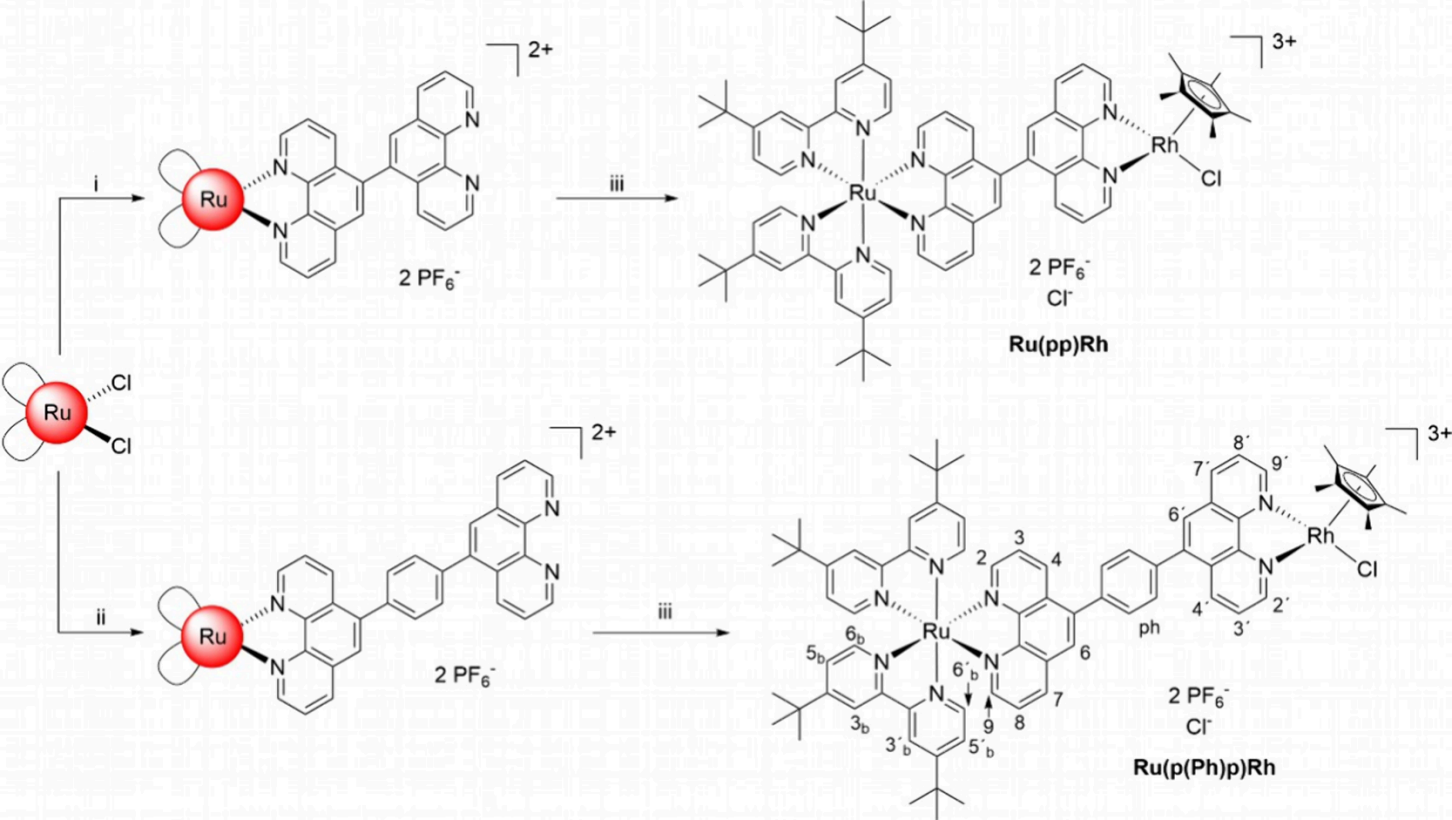
Synthesis of the Dinuclear **Ru(pp)Rh** and **Ru(p(Ph)p)Rh** Complexes Starting with the [(tbbpy)_2_RuCl_2_] Precursor Conditions: (i) (v/v = 4:1) EtOH/H_2_O, 2 eq. pp, 10 h, reflux, NH_4_PF_6_ (96%). (ii) ethanol/ethylene glycol (v:v = 1:1), 1 eq. p(Ph)p, 130 °C, 3.5 h, NH_4_PF_6_, 63.8%. (iii) DCM, 0.5 eq. [Rh(Cp*)Cl_2_]_2_, 1–2 h, r.t., 93–97%. See also Figure 2 for the labeling of the H-positions in **Ru(p(Ph)p)Rh**.

Based on the previously published synthesis of the BLs pp and p(Ph)p, as well as its mononuclear complexes [(tbbpy)_2_Ru(pp)](PF_6_)_2_ and [(tbbpy)_2_Ru(p(Ph)p)](PF_6_)_2_, the respective mononuclear Ru-complexes (see Scheme 1, steps (i) and (ii)) were resynthesized by reaction of the respective ligand with [(tbbpy)_2_RuCl_2_] in high yields (64–96%).^7,22^ The mononuclear complex was then dissolved in dichloromethane (DCM), and 0.5 equiv. of [Rh(Cp*)Cl_2_]_2_ dissolved in 5–10 mL of DCM was added (for detailed description of the syntheses, see the SI).^8,16^ After stirring for 2 h, complete conversion was achieved, yielding 97% for **Ru(pp)Rh** and 93% for **Ru(p(Ph)p)Rh**. The synthesized dyads **Ru(pp)Rh** and **Ru(p(Ph)p)Rh** are very soluble in DCM and acetonitrile (MeCN) due to the lipophilic tbbpy ligands at the Ru core. Both dinuclear RuRh complexes were characterized with respect to their structural, photophysical, and photochemical properties as well as photocatalytic and thermal catalytic activity.

**Figure 2:**
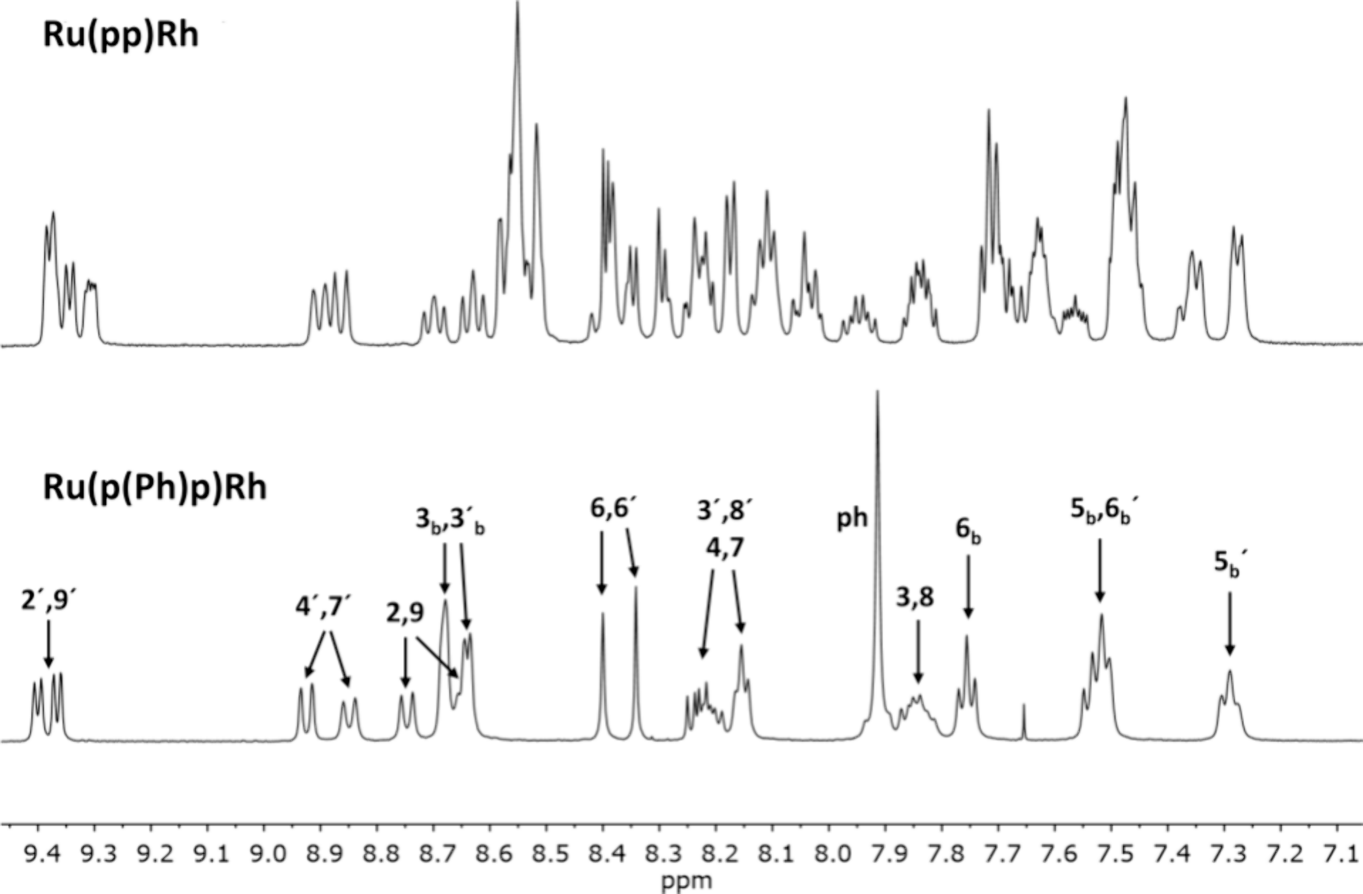
^1^H NMR spectra of the aromatic regions of **Ru(pp)Rh** (top) and **Ru(p(Ph)p)Rh** (bottom). The more complex spectrum of **Ru(pp)Rh** can be explained by the formation of four diastereomers due to the nearly perpendicular orientation of the two phenanthrolines (as for the previously reported Pt complexes; see also Table S4).^7^ Consequently, unequivocal signal assignment was possible only for **Ru(p(Ph)p)Rh**.

As shown in Figure 2, ^1^H NMR spectroscopy of the newly synthesized dinuclear complexes **Ru(pp)Rh** and **Ru(p(Ph)p)Rh** reflected the different flexibilities of the BL architectures, as indicated by the different numbers of signal sets. Similar to the previously reported [(tbbpy)_2_Ru(pp)PtCl_2_](PF_6_)_2_,^7^**Ru(pp)Rh** features a nonplanar orientation of the two 1,10-phenanthroline (phen) subunits of the BL with limited interconversion of the different stereoisomers (see also Table S4 for calculated structures). These stereoisomers are also clearly visible in the nonaromatic region of the Cp*-associated methyl groups, which split into four signals of equal intensity at ca. 1.78 ppm (see Figure S5). This, as well as the respective H,H-COSY spectrum shown in Figure S8, indicates the presence of 2^2^ = 4 different diastereomers that result from the large energy barrier associated with the rotation around the C–C single bond in the pp BL (atropisomerism) and the prochiral Rh center. By integration of the individual Cp* signals, it can be shown that all four diastereomers are equally present in solution. In comparison to these findings, the dinuclear **Ru(p(Ph)p)Rh** provided only one Cp*-based singlet at 1.775 ppm. This is due to this BL’s free rotatability via the Ph spacer decreasing the potential diastereomer number to only 1 (Figure S6). This is also reflected in the much less complicated aromatic region of the ^1^H NMR spectra depicted in Figure 2.

### Photophysical Characterization

**Figure 3:**
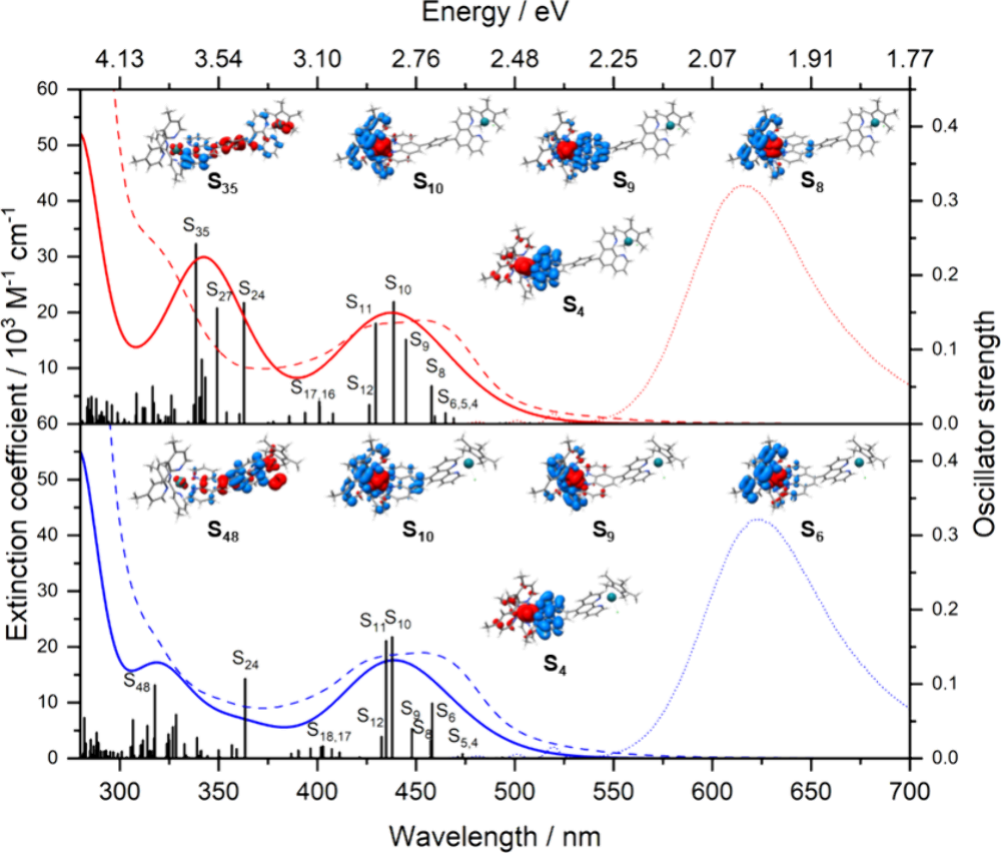
Experimental (dashed lines) and simulated (solid lines) electronic absorption spectra of **Ru(p(Ph)p)Rh** (top, red) and **Ru(pp)Rh** (bottom, blue). Charge density differences are shown for key electronic transitions, with the change in density moving from red to blue. Normalized experimental emission profiles (λ_exc_ = 450 nm) are given by the dotted lines. Spectra were obtained in aerated MeCN at room temperature (r.t.).

The ground-state absorption spectra of **Ru(pp)Rh** and **Ru(p(Ph)p)Rh** are depicted in Figure 3. Both complexes show very similar absorption properties with two prominent absorption bands at approximately 290 nm (due to ligand-based π–π* transitions) and at 450 nm (corresponding to ^1^MLCT transitions). These features agree well with the previously published spectra of Ru polypyridine complexes, e.g., [(tbbpy)_2_Ru(pp)]^2+^ and [(tbbpy)_2_Ru(p(Ph)p)]^2+^,^7,22−24^ but also with simple benchmark systems such as [Ru(phen)_3_]^2+^ or [Ru(bpy)_2_(dppz)]^2+^.^25,26^ Furthermore, these findings are consistent with the simulated electronic absorption spectra obtained at the TDDFT level of theory (see the Supporting Information for details regarding the employed computational setup) and result from the twisted nature of the pp and p(Ph)p BLs that prohibit ^1^MLCT delocalization over the whole BL (see Tables S3 and S4). As shown in Tables S3 and S4, as well as Figure 3, the 450 nm absorption band is composed of multiple MLCT transitions into S_6_–S_12_ involving π* acceptor orbitals of both tbbpy and phen moieties. However, the lowest-lying singlet transitions associated with S_1_–S_5_ have very weak oscillator strengths and thus do not contribute significantly to the absorption profile.^27^ In addition to the ^1^MLCT absorption maxima, the extinction coefficients are almost identical for **Ru(pp)Rh** (λ_max_ = 451 nm; ε_max_ = 19.0 × 10^3^ M^–1^ cm^–1^) and **Ru(p(Ph)p)Rh** (λ_max_ = 453 nm; ε_max_ = 18.6 × 10^3^ M^–1^ cm^–1^) and very similar to those of the respective mononuclear counterparts, e.g., ([(tbbpy)_2_Ru(pp)]^2+^ (λ_max_ = 454 nm; ε_max_ = 18.7 × 10^3^ M^–1^ cm^–1^) and [(tbbpy)_2_Ru(p(Ph)p)]^2+^ (λ_max_ = 455 nm; ε_max_ = 18.3 × 10^3^ M^–1^ cm^–1^)^7,22^ (see Table 1 for details). An additional shoulder is visible in the experimental absorption spectrum of **Ru(p(Ph)p)Rh** at 320 nm, which does not appear in that of **Ru(pp)Rh**. This shoulder may be attributed to a population of states S_24_, S_27_, and S_35_, which involve ligand-centered (LC) transitions of the BL, as well as MLCT contributions from the Rh(III) center to the adjacent phen acceptor (Tables S3 and S4). These transitions, while still present in **Ru(pp)Rh** (e.g., into S_48_), have weaker predicted oscillator strengths.

**Table 1:** Summary of Photophysical Data of **Ru(pp)Rh** and **Ru(p(Ph)p)Rh** as well as the Literature-Known Compounds [Ru(tbbpy)_3_]^2+^, [(tbbpy)_2_Ru(phen)]^2+^, [(tbbpy)_2_Ru(pp)]^2+^, and [(tbbpy)_2_Ru(p(Ph)p)]^2+^ in MeCN

**complex**	**λ**_**max,abs**_**[nm], (ε,[10**^**3**^ **M**^**–1**^ **cm**^**–1**^**])**	**λ**_**max,emi**_**[nm]**	**ϕ**_**em**_	**ϕ**_**em**_^**Ar**^	**τ**^**O**_**2**_^**[ns]**	**τ**^**Ar**^**[ns]**
**Ru(pp)Rh**	451 (18.97)	624	0.01	0.05	118	648
[(tbbpy)_2_Ru(pp)]^2+^^7^	454 (18.68)	618	0.01	0.20	127	2000
**Ru(p(Ph)p)Rh**	453 (18.60)	615	0.01	0.15	109	1600
[(tbbpy)_2_Ru(p(Ph)p)]^2+^^7,22^	455 (18.33)	617	0.01	0.19	110	1850
[Ru(tbbpy)_3_]^2+^^28,29^	458 (17.34)	613	0.01	0.05	107	730
[(tbbpy)_2_Ru(phen)]^2+^^30^	454 (16.00)	610	0.01	0.14	211	1420

The emission maximum in **Ru(p(Ph)p)Rh** (λ_max,emi_ = 615 nm) coincides—within the experimental accuracy—with the emission of [(tbbpy)_2_Ru(p(Ph)p)]^2+^. However, the emission shifts to longer wavelengths for **Ru(pp)Rh** (λ_max,emi_ = 624 nm), which appears to be slightly more red-shifted than the spectrum of the mononuclear counterpart [(tbbpy)_2_Ru(pp)]^2+^ (λ_max,emi_ = 618 nm), likely due to the shorter BL and the thus transmitted stabilizing effect due to the electrostatic interactions of the Rh(III) cation center on the emissive state. Furthermore, according to theoretical calculations, these results indicate the presence of a low-lying ^3^MC state at the Rh center (see Figure S24).

The luminescence loss upon Rh center integration is more pronounced when the complex is studied under an argon atmosphere. The introduction of the Rh(III) center significantly decreases the emission quantum yield of the mononuclear complexes under an argon atmosphere. The quantum yield drops from 20% for [(tbbpy)_2_Ru(pp)]^2+^ to 5% for **Ru(pp)Rh**, while for the phenyl-bridged systems, the decrease is less pronounced, from 19% for [(tbbpy)__2__Ru(p(Ph)p)]^2+^ to 15% for **Ru(p(Ph)p)Rh** (see Table 1). This trend is consistent with the observed emission lifetimes. For the pp-based system, the lifetime decreases significantly from 2000 ns (mononuclear) to 648 ns (dinuclear). This indicates additional nonradiative deactivation pathways introduced by the Rh center, which compete with the radiative decay of the ^3^MLCT. In particular, theoretical calculations (see Figure S24) reveal the presence of a low-lying ^3^MC_Rh_ state, which provides a viable deactivation pathway, especially in the pp-based system having a stronger stabilized ^3^MC_Rh_ state than **Ru(p(Ph)p)Rh**.

When the dinuclear complexes are compared, both **Ru(pp)Rh** and **Ru(p(Ph)p)Rh** exhibit similar spectral features for the ^3^MLCT emissive state in aerated MeCN, as shown in Table 1. Under an argon atmosphere, however, the emission lifetimes differ notably. The longer lifetime of **Ru(p(Ph)p)Rh** (1600 ns vs 648 ns for **Ru(pp)Rh**) can be attributed to the increased bridging ligand length in **Ru(p(Ph)p)Rh** and the energy of the ^3^MC_Rh_ state (see Figure S24). Indelli et al. observed in RuRh dyads with phenyl spacers, [(ttpy)Ru(tpy(Ph)_*n*_tpy)Rh(ttpy)]^5+^ (*n* = 0, 1, 2) (ttpy = 4′-*p*-tolyl-2,2′:6′,2″-terpyridine, tpy = 2,2′:6′,2″-terpyridine) where eT was observed only for the complex with *n* = 0, the formation of a triplet metal-to-metal charge transfer state.^4^ They attributed this to the large electronic factor and a smaller outer-sphere reorganizational energy.

**Figure 4:**
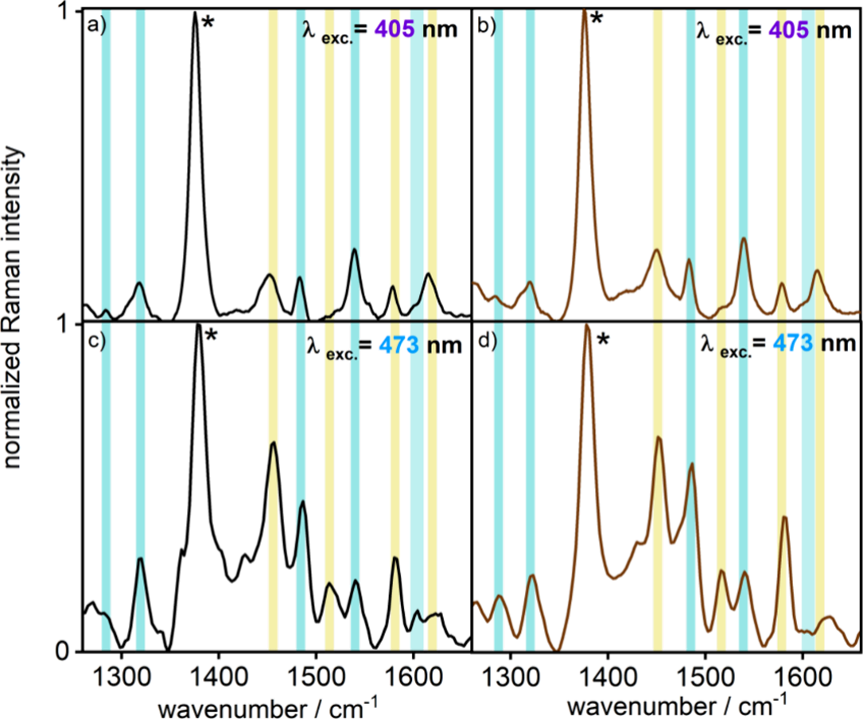
RR spectra of **Ru(p(Ph)p)Rh** in aerated MeCN excited at 405 nm (a) and 473 nm (c). The rR spectra of **Ru(pp)Rh** in aerated MeCN excited at 405 and 473 nm are depicted in panels (b) and (d), respectively. Solvent bands are marked with asterisks, and the spectra are normalized to the solvent band at 1374 cm^–1^. Characteristic Raman modes are assigned to tbbpy (light blue) and phen (yellow).

To obtain insights into the structure of the Franck–Condon-point of the complexes, resonance Raman (rR) spectroscopy is employed.^31^ Excitation wavelengths of 405 and 473 nm yield rR spectra with similar bands for both **Ru(p(Ph)p)Rh** and **Ru(pp)Rh** (see Figure 4). The mononuclear parts of these complexes have been studied and characterized previously.^7^ The similarity of the spectra shown in Figure 4 suggests no significant alteration in the nature of the initially excited state on variation of the BL, as anticipated from the very similar ^1^MLCT absorption bands in Figure 3 as well as the twisted structures of both BLs, pp and p(Ph)p, restricting ^1^MLCT distribution onto the Ru-bound phen spheres (Tables S3 and S4). On 405 nm excitation **Ru(p(Ph)p)Rh** shows bands attributed to vibrations of the tbbpy ligands at 1284, 1320, 1484, 1540, and 1605 cm^–1^ and, thus, indicates a significant involvement of the orbitals centered on the tbbpy ligands in the ^1^MLCT transitions.^16^ However, the MLCT is not exclusively centered on the tbbpy ligands, as other prominent bands in the rR spectra, such as those at 1451, 1581, and 1620 cm^–1^, which are characteristic vibrations of the phen ligand, are also enhanced.^16^ For **Ru(pp)Rh**, the characteristic tbbpy bands are observed at 1286, 1320, 1484, and 1540 cm^–1^ with respective phenanthroline bands at 1451, 1579, and 1615 cm^–1^. The simulated rR spectra of both complexes are given in Figure S23 and show good correlation with the experimental spectra obtained with 405 nm excitation. Enhancement of both tbbpy- and phen-based modes is consistent with the population of both ^1^MLCT_tbbpy_ and ^1^MLCT_phen_ states, with the strongly absorbing transitions associated with S_9_–S_12_ dominating the resonance Raman spectrum.

When the excitation wavelength is shifted to 473 nm, there is an increased enhancement of phen-localized modes. Furthermore, additional features at 1513 cm^–1^ (accompanied by a weak shoulder at 1520 cm^–1^) and 1517 cm^–1^ for **Ru(p(Ph)p)Rh** and **Ru(pp)Rh,** respectively, are observed. The rR spectra of similar Ru(II) complexes containing the phen ligand also reveal the presence of a mode in this region (1512–1516 cm^–1^), which was assigned as a Ru(II) coordinated phen-based vibration.^16,32−34^ These findings are consistent with the greater contributions of ^1^MLCT_phen_-localized excited states in this low energy region. The simulated rR spectra at 473 nm excitation for both complexes are not well matched with the experimental data. As mentioned previously, the TDDFT-predicted oscillator strengths for the excited states in this low energy region are seemingly underestimated. Hence, in the sum-over-states approach, considering only FC (A-term) scattering, the higher-lying states whose population is electric-dipole-allowed dominate the simulated spectrum. To better simulate the electronic absorption spectrum and consequently the resonance spectra incorporating these low energy, weakly allowed transitions, Herzberg–Teller (B-term) scattering, which involves vibronic coupling of the resonant state to another excited state, may need to be considered. However, such calculations would require investigation of the displacement of the potential energy surfaces along these normal modes of vibration and is beyond the scope of the present contribution.^35,36^ The excitation wavelength-dependent changes in the rR spectra of both **Ru(p(Ph)p)Rh** and **Ru(pp)Rh** indicate that photoinduced electron transfer onto the phen moiety is increased at longer excitation wavelengths, while the orbitals localized on both tbbpy and phen parts of the ligands dominate the MLCT transitions excited at 405 nm.

### Photoinduced Dynamics of Ru(p(Ph)p)Rh on 400 nm Excitation in MeCN

**Figure 5:**
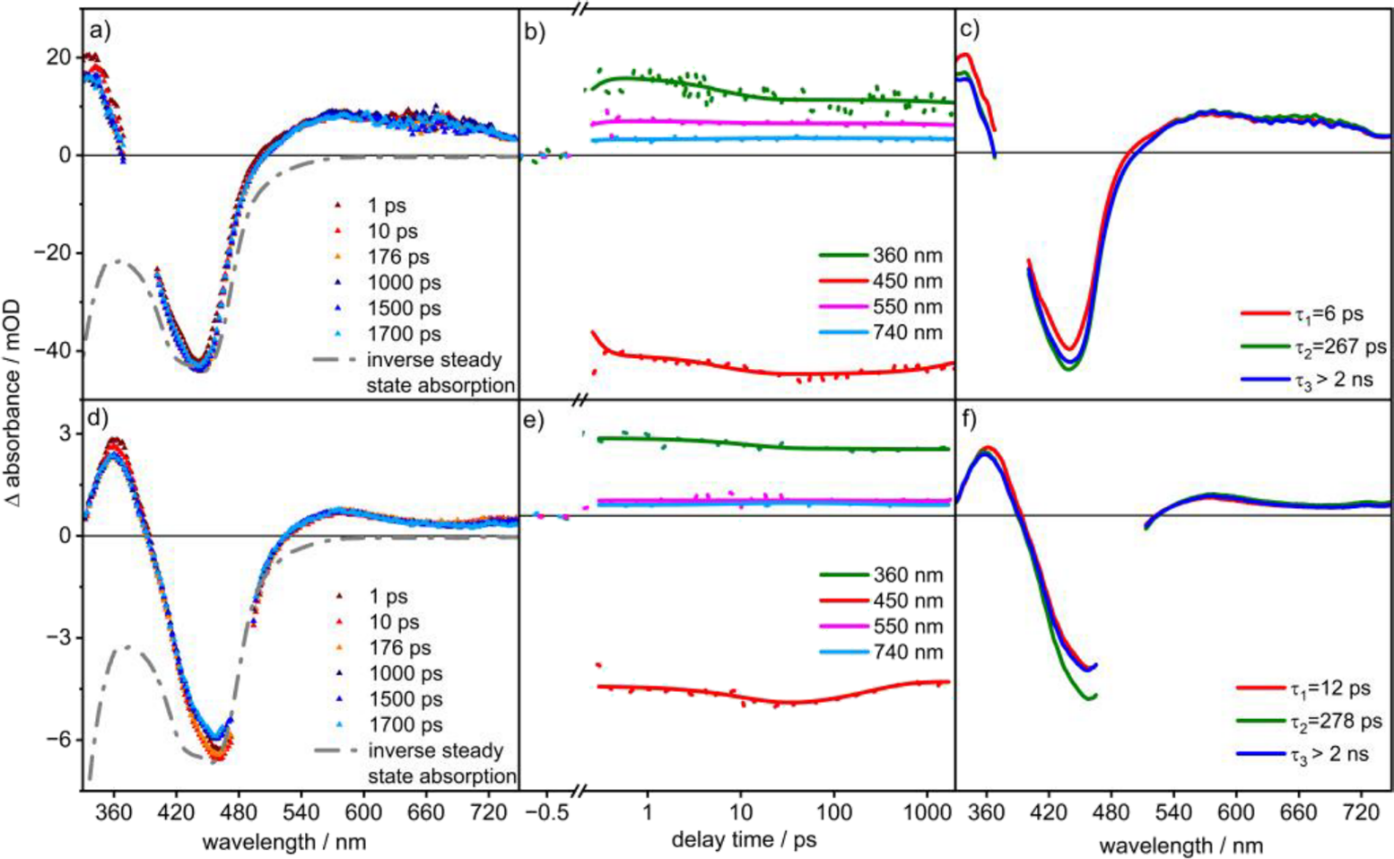
Transient absorption spectra of **Ru(p(Ph)p)Rh** in MeCN in air at excitation wavelengths of 400 nm (a) and 480 nm (d) in the wavelength range of 340–750 nm (0.4 mW). The kinetics with respect to their delay time in ps are provided in (b) and (e). (c) and (f) show the decay associated spectra (DAS) in the wavelength range of 340–750 nm.

**Figure 6:**
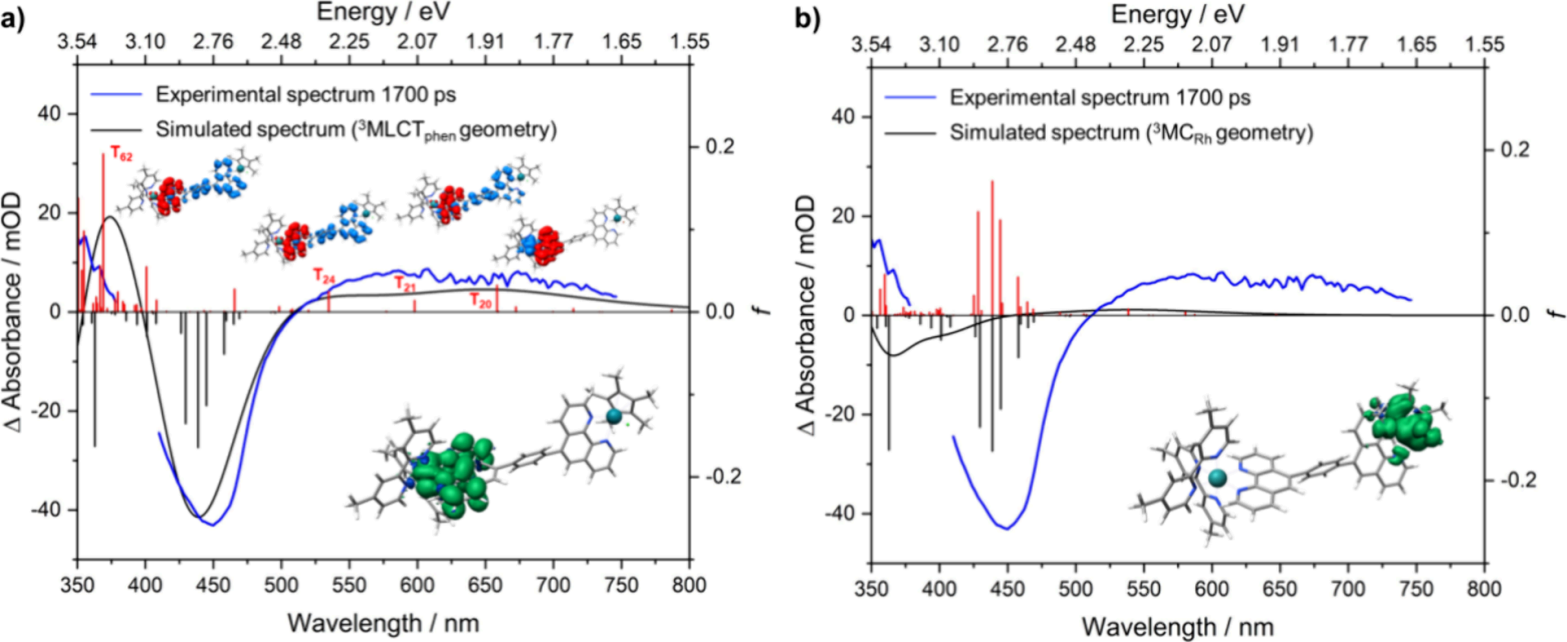
TDDFT simulated transient absorption spectra (black line) compared to experimental transient absorption spectra obtained at 1700 ps (blue line, λ_exc_ = 400 nm) for **Ru(p(Ph)p)Rh** (a, b) from DFT-optimized ^3^MLCT_phen_ or ^3^MC_Rh_ geometries. The black bars represent the singlet-to-singlet transitions obtained from the S_0_ ground-state geometry, while the red bars represent the triplet-to-triplet transitions from the respective ^3^MLCT_phen_ or ^3^MC_Rh_ optimized geometries. Electronic characters of the triplet ground state (spin density, bottom) and of key triplet–triplet transitions are visualized (charge density differences; charge transfer occurs for red to blue, top).

To investigate the excited-state dynamics of the complexes, femtosecond TA spectroscopy is employed. The TA spectra recorded on excitation at 400 and 480 nm are depicted in Figures 5 and 6, respectively. Both reveal an initial (within the first few picoseconds) increase of the excited-state absorption (ESA) at 360 nm, which stems from π–π* transitions of a reduced phen unit.^37,38^ In parallel, a strong ground-state bleach (GSB), centered at 450 nm, and a rather unstructured ESA at λ > 500 nm are observed, with the latter due to ligand-to-metal charge transfer (LMCT) transitions. These TA features recorded at short delay times, i.e., within 1 ps, thus reflect the key features also observed for prototypical complexes, e.g., [Ru(bpy)_3_]^2+^ or [Ru(phen)_3_]^2+^.^39−41^

Figure 5 depicts the TA data for **Ru(p(Ph)p)Rh** in aerated MeCN on excitation at 400 nm. In the first 10 ps after photoexcitation, the ESA band at 605 nm slightly increases, while both the ESA signal at 360 nm and the GSB signal decrease in parallel. After these initial changes, the intensity of the long-wavelength ESA, reflecting the population of a ^3^MLCT state, remains constant within the experimental window of 2 ns. The experimental ESA band at 605 nm is also consistent with the simulated ESA of a ^3^MLCT_phen_ state, as illustrated by the TDDFT-predicted spin- and dipole-allowed triplet–triplet transitions in the visible region, obtained from a DFT-optimized ^3^MLCT_phen_ geometry (Figure 6a and Figure S25). A DFT-optimized, low-lying ^3^MC state localized over the Rh(III) center was also obtained (Figure S24), which may provide another deactivation pathway to the ground state. However, as shown in Figure 6b, unlike that of the ^3^MLCT_phen_, the simulated TA spectrum from such a ^3^MC state shows minimal ESA in the visible region, and therefore, its population may be difficult to observe experimentally.

**Figure 7:**
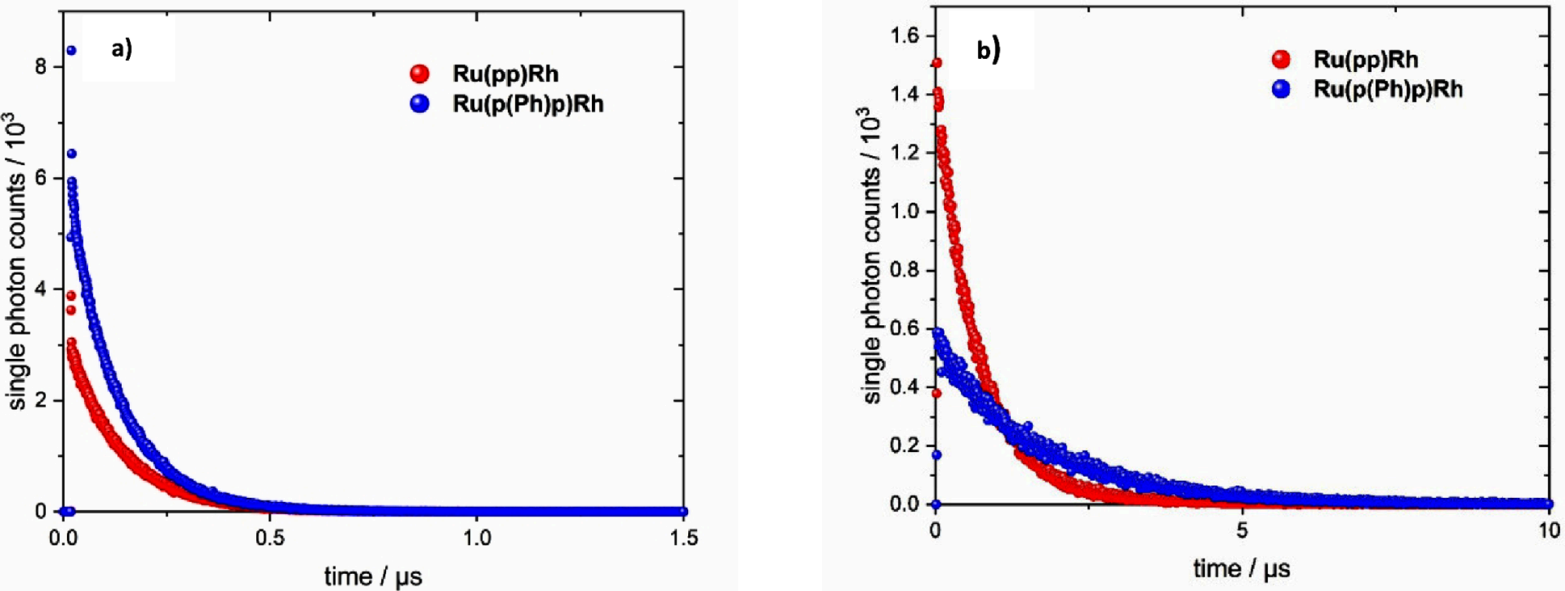
Emission decay lifetime profiles of **Ru(pp)Rh** and **Ru(p(Ph)p)Rh** in air (a) or Ar-saturated MeCN (b). Both complexes were excited at 451 nm.

**Figure 8:**
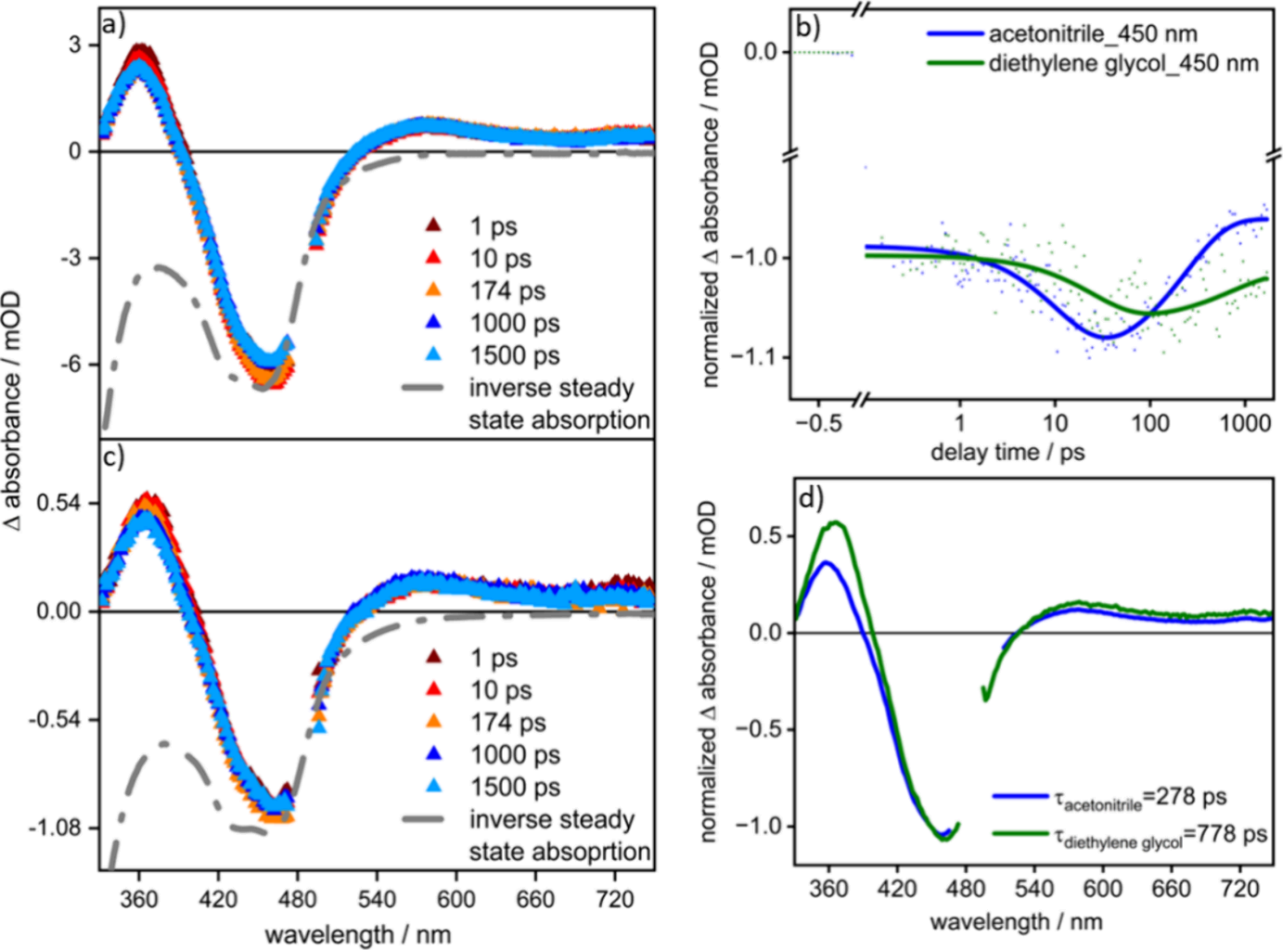
(a) and c) Transient absorption spectrum of **Ru(p(Ph)p)Rh** in MeCN and DEG solvents, respectively, at λ_exc_ = 480 nm in the wavelength range of 340–750 nm. (b) shows the comparison of the kinetics at 450 nm wavelength with respect to their delay time in ps in different solvents. (d) Comparison of decay associated spectra (DAS) in the wavelength range of 340–750 nm at τ_2_ in MeCN and DEG solvents.

An overall decay of the long-lived ^3^MLCT state is observed in the nanosecond emission measurements (see Figure 7), yielding an overall lifetime of the (emissive) ^3^MLCT state for **Ru(p(Ph)p)Rh** of about 100 ns in aerated MeCN. Kinetic analysis of the TA data by global fitting reveals two characteristic time constants and one long-lived component. The characteristic time constants determined by global fitting are τ_1_ = 6 ps and τ_2_ = 267 ps, while the lifetime of the long-lived state extends beyond the experimentally accessible delay time range of 2 ns. We associated this long-lived, nondecaying component with the lowest energy ^3^MLCT state (see Figure 7 for ns time-resolved emission measurements). The 6 ps component is associated with the vibrational relaxation of ^3^MLCT following its formation, while the 267 ps time constant could be associated with the structural relaxation (conformational changes) of the vibrationally cooled ^3^MLCT state. This is consistent with minimal spectral changes in the DAS of these three time constants. Similar observations have been previously reported by Castellano et al. in their Pt(II) terpyridine and our group in the Pt(II) perylene-based complexes, where they observed time constants of τ = 170 ps and τ = 200 ps, respectively, and assigned them to solvent reorganization and/or minor structural changes in the system. Furthermore, Machura et al. have also associated the 208 ps time constant in their Re(I) complexes featuring pyridine-based ligands with conjugated aryl groups as structural reorganization within the system.^42−45^ This assignment is further substantiated by additional measurement studies in diethylene glycol (DEG) solvent (more viscous solvent than MeCN), where the second component is much slower (504 ps in DEG from 267 ps in MeCN) under 400 nm excitation (see Figure 8 and SI Figures S12 and S13 for detailed TA measurements in DEG solvent).

### Photoinduced Dynamics of Ru(pp)Rh at 400 nm Excitation in MeCN

The TA features of **Ru(pp)Rh** were recorded on excitation at 400 nm in MeCN (see Figure S11) and are compared to the respective data of **Ru(p(Ph)p)Rh** shown in Figure 5. The TA data for **Ru(pp)Rh** reveals two characteristic time constants and one long-lived component, i.e., τ_1_ = 3 ps and τ_2_ = 250 ps. The shorter BL in **Ru(pp)Rh** gives a shorter lifetime τ_1_ as compared to **Ru(p(Ph)p)Rh**. The smaller value for τ_2_ indicates that the localization of electrons onto the phen moiety is faster in **Ru(pp)Rh** than in **Ru(p(Ph)p)Rh**, while the spectral similarities indicate that similar processes take place when the two different compounds are optically excited at 400 nm.

### Impact of Excitation Wavelength

In order to evaluate the effect of the pump wavelength, when exciting an optical transition that leads to more excess electron density on the phen part of the BL, **Ru(p(Ph)p)Rh** and **Ru(pp)Rh** dissolved in aerated MeCN are excited at 480 nm. Shifting the excitation wavelength from 400 to 480 nm induces spectral changes in the TA data: The maximum negative differential absorption signal shifts by 485 cm^–1^ from 450 to 460 nm. In addition, the ESA observed at around 580 nm becomes more structured (compared to 400 nm excitation) and develops a clearly distinguishable feature at 720 nm. These spectral changes corroborate the notion that on 480 nm excitation, the nature of the populated MLCT state is changed. Similar observations have been made, e.g., by Müller et al. studying a Ru(II) thiazole π-extended dipyridophenazine complex.^46^ For both complexes, a biexponential kinetics describes the buildup of a long-lived state (see Figures S11 and S12). The characteristic time constants are τ_1_ = 12 ps, τ_2_ = 278 ps for **Ru(p(Ph)p)Rh** and τ_1_ = 9 ps, τ_2_ = 277 ps for **Ru(pp)Rh**. In particular, the values obtained for τ_1_ are longer than those obtained on 400 nm excitation.

This might be rationalized by a reduced amount of excess vibronic energy being placed into the molecules, which, in turn, slows the charge localization on the phen moiety of the BL. Our results indicate no changes in the ESA at 360 nm, which is the characteristic band of reduced phen. This band remains intense and does not diminish or broaden on the introduction of Rh, substantiating the ^3^MLCT state being restricted to the Ru-bound phen sphere. The spectral changes in this feature could have indicated a delocalized charge intensity toward the Rh(III) center, which was observed earlier by our group in [(tbbpy)_2_Ru(tpphz)Rh(Cp*)Cl]^3+^ systems.^7^ The broad ESA wavelength above 500 nm, which determines the ^3^MLCT state of [(bpy)_2_Ru(phen)]^2+^, does not change on Rh(III) introduction at both excitation wavelengths.^45^ Thus, irrespective of the excitation wavelengths, the TA data do not reveal any sign of light-driven reduction of the respective Rh(III) centers, which is ascribed to the twisted geometry of both BLs (see Tables S3 and S4).

### Viscosity-Dependent fs-TA at 480 nm Excitation

Based on the free rotatability of the p(Ph)p BL on the ^1^H NMR time scale (see Figure 2), solvent viscosity-dependent TA studies were performed using MeCN (η = 0.37 mPa·s at 25 °C, ε = 37.5) and diethylene glycol (DEG) (η = 30 mPa·s at 25 °C, ε = 31.69). The resultant data for **Ru(p(Ph)p)Rh** are presented in Figure 8. While the spectral shape of the differential absorption signal is not impacted by the choice of solvent, the excited-state relaxation kinetics are (see Figure 8b and SI, i.e., Figures S12 and S13). The time constant τ_1_ increases from 12 ps in MeCN to 28 ps in DEG, and τ_2_ also increases from 275 ps in MeCN to 778 ps in DEG. The kinetic analysis of the TA for **Ru(p(Ph)p)Rh** in DEG yields, as before, two distinct kinetic processes, characterized by time constants τ_1_ = 28 ps and_,_ τ_2_ = 778 ps in addition to a long-lived component (see Figure 8). The more than 2-fold increased value of τ_1_, which is 12 ps in MeCN and 28 ps in DEG, indicates that the process of vibrational cooling is also controlled by solvent viscosity. With the ca. 3-fold increase of τ_2_, when changing MeCN for DEG, minor changes in the corresponding DAS spectra are observed: (i) the amplitude at 360 nm appears higher in DEG compared to MeCN, and (ii) the DAS spectrum appears overall red-shifted by about 800 cm^–1^ (see Figure 8d). While the increase in τ on increasing the solvent’s viscosity indicates some structural rearrangements being associated with the corresponding kinetic process, the spectral shifts indicate that the excited-state dipole moment of the complex changes on ligand planarization by rotation around the phen–Ph single bond.^44^ Hence, the different solvent polarities cause the observed spectral shifts.

The effect of solvent polarity on the TA data of **Ru(pp)Rh** resembles the aforementioned changes in **Ru(p(Ph)p)Rh**. As summarized in Figures S12 and S13, **Ru(pp)Rh** exhibits a slower excited-state decay in DEG compared to MeCN, despite no noticeable spectral changes when the solvent is altered. This behavior may be attributed to the restricted rotation of the shorter pp BL, which limits stereoisomer interconversion, as observed in the ^1^H NMR signals (see Figure S6). A global analysis of the TA data recorded in DEG reveals characteristic lifetimes τ_1_ = 24 ps and τ_2_ = 704 ps in addition to a long-lived component. However, compared to **Ru(p(Ph)p)Rh**, the τ_2_ value of **Ru(pp)Rh** in DEG is smaller, which might be linked to the smaller metal-to-metal distance in **Ru(pp)Rh**. As already described above, this second component (τ_2_) describes the structural relaxation of the vibrationally cooled ^3^MLCT state, which might be more energy demanding for larger BLs (for comparison of the data and more details related to kinetics and spectra, see supplementary Figures S12 and S13).

### Photostability

To get an idea of the long-term performance of the two dyads during later photocatalytic experiments, the photostability of **Ru(pp)Rh** and **Ru(p(Ph)p)Rh** was also investigated. For this, two samples of each compound with an optical density of 0.19 at 450 nm were prepared and irradiated for defined time intervals with one LED stick from the bottom of a standard 1 cm × 1 cm cuvette in aerated MeCN (470 ± 20 nm, 45 ± 5 mW·cm^–1^; see Figure S1). The light-induced changes were followed by recording the UV/vis absorption spectra during irradiation (Figures S14 and S15). By calculating the loss of absorbance at the absorption maxima (for **Ru(pp)Rh** at 451 nm and for **Ru(p(Ph)p)Rh** at 453 nm), the percentage degradation was determined (see Table S2). For **Ru(pp)Rh**, a higher photostability compared to **Ru(p(Ph)p)Rh** was observed. This observation can be rationalized (i) by means of the simulated energy gap between the ^3^MLCT state and the dissociative ^3^MC_Ru_ states at the Ru site, which is slightly smaller for **Ru(p(Ph)p)Rh** than for **Ru(pp)Rh** (see Figure S24) and (ii) the longer excited state lifetime of **Ru(p(Ph)p)Rh** increasing the likelihood of dissociative photochemistry (see Table 1).

### Electrochemical Characterization

**Figure 9:**
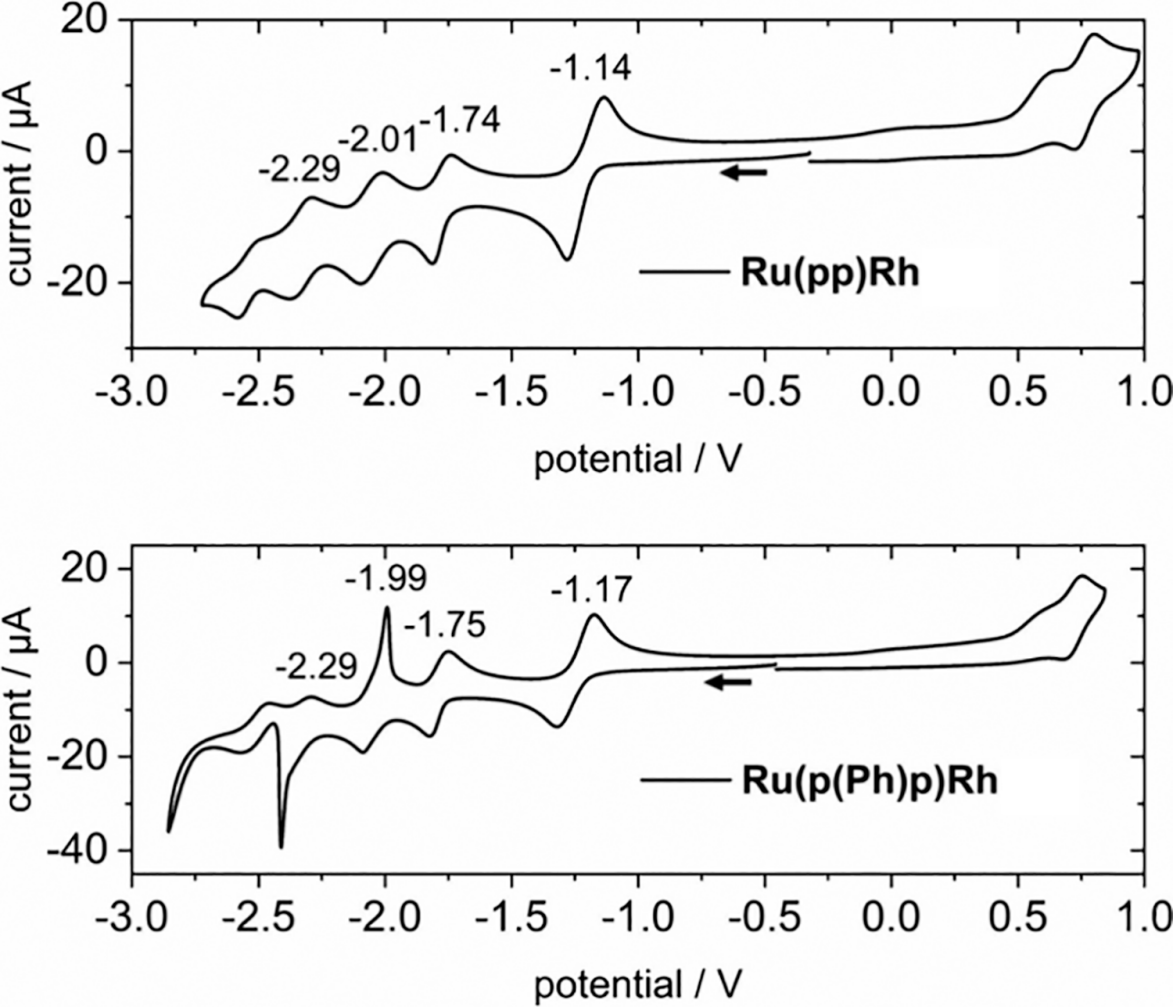
Redox properties of **Ru(pp)Rh** (top) and **Ru(p(Ph)p)Rh** (bottom) measured on a glassy-carbon working electrode (scan rate of 100 mV·s^–1^) in degassed MeCN containing 0.1 M *n*Bu_4_NPF_6_ as electrolyte. Further, a Ag/AgCl reference electrode and a Pt wire counter electrode were used. All measured potentials were converted to the Fc^+^/Fc redox couple.

To rationalize later photocatalytic activity differences (vide infra), the redox properties of the newly synthesized **Ru(pp)Rh** and **Ru(p(Ph)p)Rh** complexes were analyzed in degassed MeCN containing 0.1 M *n*Bu_4_NPF_6_ as electrolyte and referred to the Fc^+^/Fc redox couple as shown in Figure 9. Both complexes showed the distinct Rh(III)/Rh(I) redox event at −1.14 V vs Fc^+^/Fc for **Ru(pp)Rh** and −1.17 V vs Fc^+^/Fc for **Ru(p(Ph)p)Rh**, in good agreement with the previously published [(tbbpy)_2_Ru(p(C_2_)p)Rh(Cp*)Cl]^3+^.^16^ The quasi-identical redox potential of the CAT thus excludes a Rh-chemistry-responsible variation of photocatalytic activity but rather limits this discussion to the photochemistry of the BL-differing dyads. At approximately −1.7 V vs Fc^+^/Fc, the reduction of the bridging ligands takes place while the other two redox-events at approximately −2 and −2.29 V vs Fc^+^/Fc can be attributed to the reduction of the tbbpy ligands.^16^ In good agreement with the previously published RuRh complexes, the Ru(III)/Ru(II) oxidation can be observed at ca. 0.8 V vs Fc^+^/Fc. Furthermore, the chloride is irreversibly oxidized at approximately 0.6 V vs Fc^+^/Fc. The sharp spikes for **Ru(p(Ph)p)Rh** at ca. −2.4 V (reduction) and −2.0 V (reoxidation) might be due to a deposition of the complex on the electrode.

### Thermally Driven NAD^+^ Reduction to NADH

To evaluate the catalytic activity of the newly synthesized **Ru(pp)Rh** and **Ru(p(Ph)p)Rh**, it is important to determine if (i) in the presence of a sacrificial electron donor (SED) an eT from PS to CAT (e.g., via reductive quenching by SED) is feasible and (ii) if the Rh(III) center is in general active toward NAD^+^ to NADH reduction. To evaluate the latter, 5 μM **Ru(pp)Rh** or **Ru(p(Ph)p)Rh** was incubated with 50 mM NaHCO_2_ and 250 μM NAD^+^ in 9:1 (v:v) H_2_O:MeCN (for detailed description, see the SI).

As shown in Figures S19–S21, both catalytic systems act in the same manner with typical catalytic activity for NADH formation (TON = 39 after 50 min). This clearly indicates that both newly investigated BLs, i.e., pp and p(Ph)p, are able to bind an identically behaving active Rh center as could be inferred by the virtually identical Rh(III/I) reduction potentials of **Ru(pp)Rh**, **Ru(p(Ph)p)Rh** and previously reported dinuclear systems (see Figure 1). Consequently, any differences in photocatalytic activity can be correlated to the photophysics-originating photochemical differences rather than a different ground-state chemistry of the Rh centers.

### Photochemical Reduction of Rh(III) to Rh(I) in the RuRh Dyads

Based on these results on thermally driven NADH formation, and despite the results of the TA data indicating no intramolecular eT from PS to CAT in pure solvent, it was still analyzed whether light-driven reduction of the Rh CAT is feasible in the presence of a SED. For this, a 10 μM solution of either **Ru(pp)Rh** or **Ru(p(Ph)p)Rh** containing 0.12 M triethylamine (TEA) in 9:1 (v:v) H_2_O:MeCN mixture was prepared. As already known for other dinuclear RuRh photocatalysts (see top row in Figure 1), reduction of the Rh(III) center to Rh(I) causes an increase in absorption at ca. 680 nm.^16^ For this, the deaerated samples were irradiated (for setup, see Figure S3) with one LED stick (470 ± 20 nm, 45 ± 5 mW·cm^–1^). Time-dependent measurement can reveal the kinetics for the formation of the catalytically active, 2-fold reduced Rh(I) species. To analyze concentration-dependent effects, the same compounds were also investigated at 20 μM.

As shown in Figure S22, a faster formation of the Rh(I) species is observed in the case of **Ru(p(Ph)p)Rh** (τ_1/2_ of 32 s (10 μM) and 78 s (20 μM); τ_1/2_ represents the time at which the Rh(I) signal reaches half of its maximum intensity) compared to **Ru(pp)Rh** (τ_1/2_ of 56 s (10 μM) and 90 s (20 μM)). The difference between **Ru(pp)Rh** and **Ru(p(Ph)p)Rh** is likely linked to the prolonged lifetime of the latter (see Table 1), making a reductive-quenching-based Rh(I) formation more efficient. Based on previous reports showing a correlation between the rate of Rh(I) formation and the rate of photocatalytic NADH formation,^16^ as well as the τ_1/2_ ratio of the two RuRh dyads of ca. 2, a 2-fold higher activity for **Ru(p(Ph)p)Rh** on the light-driven NAD^+^ reduction was anticipated (and observed; see next section). Interestingly, whereas **Ru(p(Ph)p)Rh** shows a dilution-induced (20 μM → 10 μM) acceleration of Rh(I) formation by 59%, the acceleration for **Ru(pp)Rh** is only 38%. A dilution-induced acceleration of the Rh(I) formation has already been observed for the efficient alkynyl-bridged photocatalyst shown in the center of the top row in Figure 1.^16^ We ascribe this phenomenon to a photonic limitation at higher concentrations for all complexes, generating the Rh(I) state preferably via two successive intramolecular electron transfers. These systems benefit from the dilution-induced increase of the photon number per molecule, whereas other Ru(BL)Rh systems might prefer forming Rh(I) via a disproportionation reaction^47^ or whether the influence of protonation of the BL (shown to be very important for tpphz^10^ and also feasible for the triazole in Figure 1 top right) impacts the Rh(I) formation processes. Obviously, for these weakly communicating Ru(BL)Rh systems, they do benefit less or not at all from dilution. Furthermore, as the dilution-induced acceleration of Rh(I) formation for **Ru(p(Ph)p)Rh** is larger than that for **Ru(pp)Rh**, it is concluded that **Ru(p(Ph)p)Rh** activates the Rh center to a higher extent via two successive intramolecular electron transfers compared to **Ru(pp)Rh**, which relies more strongly on a disproportionation mechanism.

### Light-Driven NAD^+^ Reduction to NADH

**Figure 10:**
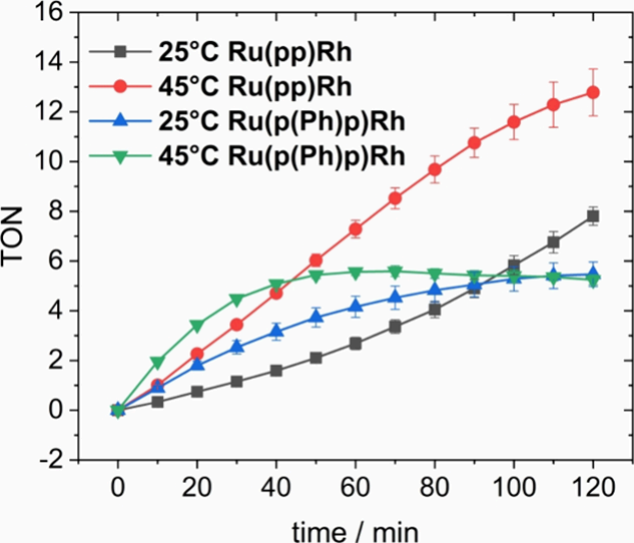
TON of light-driven NAD^+^ to NADH reduction for **Ru(pp)Rh** and **Ru(p(Ph)p)Rh** at 25 and 45 °C in 2:1 (H_2_O:MeCN, *V*_total_ = 3 mL) with 5 μM RuRh photocatalyst, 250 μM NAD^+^, 0.12 M TEA, 0.10 M NaH_2_PO_4_, and irradiated with one LED stick (470 ± 20 nm, 45 ± 5 mW·cm^–1^). The data represent average values of *n* = 3–5 measurements; error bars represent standard deviations.

In order to evaluate the capacity of the two RuRh dyads to perform light-driven catalysis, mixtures of **Ru(pp)Rh** or **Ru(p(Ph)p)Rh** (5 μM), 0.12 M TEA, 0.10 M NaH_2_PO_4_, and 250 μM NAD^+^ as a substrate for the catalysis in 2:1 (v:v) deaerated H_2_O:MeCN were prepared. As photocatalytic NADH formation has already been shown to be positively influenced by temperature,^16^ it was performed at 25 and 45 °C (see Figure 10 and Figure S16). To provide a constant temperature, the reaction setup shown in Figure S2 was used. Irradiation was achieved by utilization of one LED stick (470 ± 20 nm, 45 ± 5 mW·cm^–1^) through the bottom of the cuvette. In analogy to thermal catalysis, spectral changes at 340 nm in the steady-state absorption measurement were characteristic of NADH formation.

Especially, at the start of the experiment, higher photocatalytic activity was observed at higher temperatures (i.e., higher TOFs, see Figures S16–S17). **Ru(p(Ph)p)Rh** proved to be rather active at 45 °C at the beginning. However, its lower photostability also under photocatalytic conditions (Figure S18) limits the overall TON to ca. 5.8. Interestingly, reduction of the temperature to 25 °C not only slows down decomposition but also prolongs catalytic activity from ca. 40–90 min. However, the amount of produced NADH was identical compared to 45 °C. In both cases, the limiting factor was determined to be the low photostability under catalytic conditions, in accordance with the studies in pure MeCN (see Figure S15 and Table S2). In line with previous studies on the correlation between the rate of Rh(I) formation and (initial) photocatalytic activity,^16^**Ru(p(Ph)p)Rh** is more active than **Ru(pp)Rh** before its more emphasized photodegradation sets in. The anticipated ca. 2-fold increased activity of **Ru(p(Ph)p)Rh** over **Ru(pp)Rh** is best matched after 10 min of irradiation (see Figure S17) before rapid photodegradation of the more active dyad has a remarkable influence on the NADH formation rate. Overall, **Ru(pp)Rh** reaches a higher overall TON after 2 h than **Ru(p(Ph)p)Rh** (ca. 13 at 45 °C and ca. 8 at 25 °C).

## Conclusions

This study investigates the effect of a phenyl spacer inside a bridging ligand (BL) connecting structurally and electrochemically identical photosensitizer (PS) and catalytic center (CAT) moieties on the photophysical, photochemical, and photocatalytic properties of the resultant dinuclear complexes **Ru(pp)Rh** and **Ru(p(Ph)p)Rh**. Quantum chemical simulations and femtosecond transient absorption studies conducted at various excitation wavelengths and in different solvents reveal that electron transfer from Ru to Rh centers is hindered due to rotation of the BL (phen–Ph bond in **Ru(p(Ph)p)Rh** and phen–phen bond in **Ru(pp)Rh**), restricting ^3^MLCT delocalization to the Ru-bound phen part of the BL. Density functional theory calculations show that low-lying ^3^MC states were localized over the Rh(III) center, which provides another deactivation pathway. Nevertheless, in the presence of TEA, light-driven reduction of CAT was observed that must have proceeded via reductive quenching of the long-lived ^3^MLCT state. As the excited state lifetime of **Ru(p(Ph)p)Rh** proved to be significantly longer than that of the shorter **Ru(pp)Rh** complex, a more rapid electron collection at the CAT site was observed for **Ru(p(Ph)p)Rh**. This also translated into higher photocatalytic activity in the initial phase of catalysis. However, the higher photolability of **Ru(p(Ph)p)Rh** due to a lower ^3^MLCT/^3^MC-at-Ru gap resulted in an overall less efficient NAD^+^ photoreduction. This study thus highlights the complex interplay between photophysics and photostability in photocatalytic output in supramolecular photocatalysts even if the change in the BL is as subtle as the introduction of a phenyl spacer that did not change the ground-state redox properties of PS and CAT. As no intramolecular electron transfer took place in the presented RuRh dyads, photocatalysis could be improved by coupling the pp and p(Ph)p BLs to a stronger oxidizing PS, thereby potentially accelerating reductive quenching, electron collection at CAT, and likely also photostability.

## Experimental Section

### Chemicals

The ligands pp and p(Ph)p, the precursor [Ru(tbbpy)_2_Cl_2_], and the mononuclear complexes [(tbbpy)_2_Ru(pp)](PF_6_)_2_ and [(tbbpy)_2_Ru(p(Ph)p)](PF_6_)_2_ were synthesized according to previously published literature procedures.^7,22,48^ All reagents were purchased from either VWR, Sigma-Aldrich, or abcr and used without further purification. Assignment of the ^1^H NMR signals was achieved by using 2D-NMR spectroscopy. For detailed synthesis, see the Supporting Information.

### Spectroscopy

#### Steady-State UV/Vis Absorption and Emission Spectroscopy

All absorption and emission spectra were recorded on a Jasco V-670 UV/vis spectrophotometer or a Jasco FP-8500 spectrofluorometer using a 10 mm × 10 mm quartz glass cuvette. All absorption spectra were recorded under aerated conditions in spectroscopic grade MeCN. Nanosecond measurements were performed in both aerated and argon-saturated MeCN, as diffusion-controlled processes take place on a ns time scale and molecular oxygen (O_2_) is a highly efficient quencher of triplet state formation in the excited state.^49^

#### Transient Absorption Spectroscopy

The femtosecond transient absorption spectroscopy (fs-TA) measurements were obtained using the already reported setup, which was reported previously.^50,51^ The measurements done in this setup were based on a Ti-Sapphire (Astrella, Coherent, USA), which generates ultrashort pulses at 800 nm centered with a repetition rate of 1 kHz. The broadband supercontinuum white light ranging from 300 to 700 nm was generated by the first part of the laser focusing on a rotating CaF_2_ crystal. This resultant supercontinuum was then split into two parts, namely, one as a reference pulse and the other as a probe pulse. To generate a pump pulse, the second fraction of the laser pulse was directed to an optical parametric amplifier (TOPAS prime, Light Conversion, Lithuania). The mutual polarization of pump- and probe-pulses was set to magic angle, and the pulses were overlapped at the sample position. In this study, the pump pulses were set as 400 and 480 nm. The solution samples were prepared in a 1 mm path length quartz cuvette to yield an optical density ranging of 0.2–0.4 at the excitation wavelengths. The power of the pump pulse was set to 0.4 μJ/pulse. UV/vis spectroscopy was measured before and after the measurements to monitor the photodegradation. The fs-TA data analysis was obtained using a KimoPack software tool.^52^ After the chirp correction, the fitting was obtained by performing the global fit in the data. The data of 150 fs was omitted to avoid coherent artifacts.^53^ Global analysis was obtained using a consecutive fitting model at a confidence level of 95%. The transient spectra, kinetics, and decay associated spectra (DAS) are shown in Figures S11–S13.

#### Resonance Raman Spectroscopy

Resonance Raman (rR) measurements were performed using an Ar-ion laser (Innova300, Coherent, USA) excited with 405 and 473 nm blue diode pumped solid state lasers (HB-Laser, Germany). The Raman scattered signals were detected in an IsoPlane 160 grating spectrometer (Princeton Instruments, USA) with a slit width of 50 μm and 1200 grooves/mm grating. The Raman signal was collected using long pass filters of 405 and 473 nm (Semrock, USA). The laser power was set to 6 mW at the sample position. The MeCN band peak at 1370 cm^–1^ was used as reference to normalize the intensities and wavenumbers for the spectrum of the samples.

### Computational Details

All quantum chemical calculations were performed using the Gaussian 16 program^54^ (B.01). The singlet ground-state geometries of **Ru(p(Ph)p)Rh**, **Ru(pp)Rh**, and [Ru(tbbpy)_2_(phen)]^2+^ were obtained at the density functional theory (DFT). The B3LYP^55,56^ functional with the split-valence def2-SVP basis set^57^ was utilized, with long-range interactions accounted for by inclusion of Grimme’s D3 dispersion correction^58^ with Beck–Johnson damping. An implicit MeCN solvent field was incorporated with the integral equation formalism, employing the SMD model.^59^ The *tert*-butyl groups of all structures were approximated as CH_3_ groups to reduce computational cost.

Time-dependent DFT (TDDFT) calculations were performed on the optimized singlet ground-state geometries using the same protocol to calculate the lowest 100 singlet states. Simulated rR spectra were calculated within the independent mode displaced oscillator model as reported previously in detail.^31,60^ In summary, simulated spectra at 473 nm included gradients obtained for excited states S_1–12_ (473 nm excitation) and states S_1–20_ and S_24_ (405 nm excitation) for both **Ru(p(Ph)p)Rh** and **Ru(pp)Rh**. Homogeneous broadening of 3000 cm^–1^ and a fwhm value for the vibrational transitions of 11 cm^–1^ were applied, with spectra scaled by 0.97 to account for anharmonicity effects and insufficient treatment of electron correlation.^61^

Triplet states of **Ru(p(Ph)p)Rh** and **Ru(pp)Rh** were also optimized. Frequency calculations were performed on all optimized geometries to verify that a minimum on the potential energy surface had been obtained. Analysis of the spin densities of the optimized triplet structures revealed the presence of triplet states with ^3^MLCT_phen_, ^3^MC_Rh_, and ^3^MC_Ru_ character.

TA spectra were simulated by subtracting the electronic absorption spectrum of the ground state (obtained from the optimized S_0_ geometry) from the electronic absorption spectrum of the selected triplet state in a 1:1 ratio. The excited-state absorption features were obtained by spin and dipole-allowed triplet-to-triplet TDDFT calculations for the lowest 200 triplet states. This procedure was performed for the optimized triplet structures of **Ru(p(Ph)p)Rh** and **Ru(pp)Rh** with ^3^MLCT_phen_ and ^3^MC_Rh_ character and then compared with experimentally obtained TA spectra. Charge density difference (CDD), molecular orbital and spin-density plots were obtained with Multiwfn.^62^

## Data Availability

The data underlying this study are openly available in Zenodo at DOI: 10.5281/zenodo.15189283.

## Supporting Information Available

Additional experimental details and methods, including photographs of experimental setup, 1D- and 2D-NMR spectra for heterodinuclear complexes, additional TA data, photostability and (photo)catalysis data for heterodinuclear complexes, detailed results of density functional theory and time-dependent density functional calculations (PDF)
