# Supplementary material for: Role of Spacers in Molecularly Linked RuRh Dyads: A Comparative Synthetic and Ultrafast Spectroscopic Investigation
Source: Inorg Chem. 2025 Apr 10;64(15):7273–85. doi: 10.1021/acs.inorgchem.4c04596 (PMC12015815; doi:10.1021/acs.inorgchem.4c04596)
Supplement: Supplementary file 1 — ic4c04596_si_001.pdf [file ic4c04596_si_001.pdf]

Supporting information

Role of spacers in molecularly linked RuRh dyads: A comparative synthetic and ultrafast spectroscopic investigation

Mohini Semwal,[+ a,b] *Martin Lämmle,*[+ c,d] *Elias H. P. Brohmer,*[a] *Steffen Volk,*[e] *Linda Zedler,*[a,b] *Stephan Kupfer,*[a] *Alexander K. Mengele,*[c] *Georgina E. Shillito,*[a] *Sven Rau*[*c] *and Benjamin Dietzek-Ivanšić*[*a,b,f]

[a] Institute of Physical Chemistry, Friedrich Schiller University Jena, Helmholtzweg 4, Jena 07743, Germany

[b] Research Department Functional Interfaces, Leibniz Institute of Photonic Technology, Albert-Einstein-Str. 9, Jena 07745, Germany

[c] Institute of Inorganic Chemistry I, Ulm University, Albert-Einstein-Allee 11, Ulm 89081, Germany

[d] Zentrum für Sonnenenergie- und Wasserstoff-Forschung Baden-Württemberg, Helmholtzstraße 8, Ulm 89081, Germany

[e] Institute of Organic Chemistry I, Ulm University, Albert-Einstein-Allee 11, Ulm 89081, Germany

[f] Center for Energy and Environmental Chemistry Jena (CEEC Jena), Friedrich Schiller University Jena, Lessingstraße 8, Jena 07743, Germany

+ These authors contributed equally to the work.

* E-Mail: sven.rau@uni-ulm.de

* E-mail: benjamin.dietzek@uni-jena.de

Table of contents

1. Instrumentation ... 3

1.1 Quantum yield determinations ... 3

1.2 Photostability measurements ... 3

1.3 Electrochemistry ... 3

1.4 Mass spectrometry ... 3

1.5 NMR experiments ... 3

1.6 Emission lifetimes ... 4

1.7 Determination of NADH formation ... 4

1.8 Time-dependent Rh(I) formation ... 5

2. Synthesis ... 6

2.1 Synthesis of [(tbbpy)$_2$Ru(pp)Rh(Cp*)Cl]Cl(PF_6)$_2$ (**Ru(pp)Rh**) ... 6

2.2 Synthesis of [(tbbpy)$_2$Ru(p(Ph)p)Rh(Cp*)Cl]Cl(PF_6)$_2$ (**Ru(p(Ph)p)Rh**) ... 6

3. Supplementary Figures ... 7

3.1 Reaction setups ... 7

3.2 NMR experiments ... 9

3.3 Mass spectrometry ... 12

3.4 Transient absorption spectroscopy ... 13

3.5 Photostability ... 15

3.6 Photocatalysis ... 17

3.7 Thermal catalysis ... 19

3.8 Rh(I) formation ... 21

3.9 Theoretical Calculations ... 22

4. References ... 30

1. Instrumentation

1.1 Quantum yield determinations

The quantum yields were determined in aerated and argon-saturated MeCN, respectively, using following equations:

$$Q_f^i = Q_f^s \cdot \left(\frac{F^i . f_s . n_i^2}{F^s . f_i . n_s^2}\right) = Q_f^s \cdot \left(\frac{F^i . f_s}{F^s . f_i}\right) \quad (1)$$

$$with f_x = 1 - 10^{-A_x}$$

Q_f^i = to be determined quantum yield, Q_f^s = quantum yield of the reference compound, A_x = $A_{s,i}$= absorption of the reference and sample respectively, f_x = absorptance, $F^{s,i}$ = area of the emission of the reference and sample respectively, $n_{s,i}$ = refractive indices of the reference and sample respectively.[1]

$$Q_x = Q_R . \left(\frac{A_R}{A_X}\right) . \left(\frac{E_X}{E_R}\right) \quad (2)$$

Q_x = to be determined quantum yield, Q_R = quantum yield of the reference compound [Ru(bpy)$_3$](PF$_6$)$_2$ ($Q_R = 0.095$),[2] A_R/A_x = respective absorption intensities at 450 nm and E_R/E_X = respective emission area after excitation at 450 nm.

The quantum yields presented in the manuscript are the average values of triplicate measurements.

1.2 Photostability measurements

All photostability measurements were performed by irradiating (one LED-stick (470 ± 20 nm, 45 ± 5 mW·cm^{-1})) the respective compound (similar optical densities (OD) with approx. 0.19 at 450 nm) in aerated acetonitrile in a 10mm x 10 mm quartz glass cuvette (see Figure S1). In defined time intervals, absorption spectra were recorded using the above-mentioned absorption setup.

1.3 Electrochemistry

All electrochemical data were recorded with an Auto lab potentiostat PGSTAT204 from Methrom using a three-electrode configuration (working electrode: glassy carbon disc with a 3 mm diameter stick, counter electrode: a Pt wire and reference electrode: non-aqueous Ag/Ag$^+$ electrode with 0.01 M AgNO$_3$ in MeCN). All experiments were conducted in dry, degassed MeCN under continuous argon flow with 0.1 M TBAPF$_6$ as supporting electrolyte. As reference, the ferrocene/ferrocenium (Fc/Fc$^+$) couple was applied, which was added to the solution after each measurement. Thus, all reported potentials were corrected against the Fc/Fc$^+$ couple. All scan rates were 100 mV/s unless otherwise noted.

1.4 Mass spectrometry

High-resolution mass spectrometry (HRMS) was perfumed using a Fourier Transform Ion Cyclotron Resonance (FT-ICR) mass spectrometer solariX (Bruker Daltonik) equipped with a 7.0 T superconducting magnet and interfaced to an Apollo II Dual ESI/MALDI source.

1.5 NMR experiments

All NMR experiments were conducted on a Bruker Ascend 400 MHz or Bruker AVANCE 400 MHz at ambient temperature. All chemical shifts (δ) are given in parts per million (ppm) using residual solvent protons (δH = 7.26 ppm and δC = 77.16 ppm for CDCl$_3$, δH = 1.94 ppm

and δC = 118.26 ppm for MeCN-d_3 and δH = 3.31 ppm and δC = 49.05 ppm for MeOD-d_4) as the internal standard.

1.6 Emission lifetimes

Fluorescence lifetimes were recorded with a DeltaPro (Horiba Scientific) using a 451 nm pulsed laser source (Class 3B laser product, <0.5 W peak in pulsed and CW mode). The Delta Pro consists of: DeltaDiode (picosecond supply) and a PDD (picosecond photon detection module). The IRF (instrument response function) was measured with LUDOX silica nanoparticles. Fits were done with the software EzTime with a mono exponential decay. Higher exponential decays were ruled out by plotting the logarithmic data (single photon counts) against the time.

1.7 Determination of NADH formation

This was done following a previously published protocol.[3]

For thermal NAD^+ reduction, 15 nmol of **Ru(pp)Rh** or **Ru(p(Ph)pRh** were transferred via a stock solution to a standard GC-vial (V_{total} = 4.5 mL). After evaporation of the solvent, the GC-vial was transferred to the glovebox where subsequently 300 µL of acetonitrile was added, followed by 2.4 mL freshly prepared 62.5 mM $NaHCO_2$-water solution (final concentration was 50 mM). After addition of 300 µL NAD^+-water solution (final concentration 250 µM), the mixture was mixed and transferred to a standard 10 x 10 mm standard quartz glass cuvette sealed with a screw cap to avoid oxygen contamination. The cuvette was placed in a temperature-controlled water bath at the required temperature. The catalytic TON was determined by the absorption band at 345 nm of the respective UV/Vis spectra at various defined time intervals by utilization of the extinction coefficient of the formed NADH (56701 $mol^{-1}\cdot cm^{-1}$).

For photocatalytic experiments, 15 nmol of **Ru(pp)Rh** or **Ru(p(Ph)p)Rh** was added to a standard GC-vial (V_{total} = 4.5 mL) via a stock solution. After evaporation of the solvent, the GC-vial was introduced into the glovebox under argon atmosphere. To the catalyst, 1 mL of degassed MeCN, 1 mL of a freshly prepared triethylamine (TEA)/NaH_2PO_4 solution in water (0.36 M/0.30 M) yielding a final concentration of 0.12 M/0.1 M and 1 mL of an NAD^+ solution (750 µM) in water yielding a final concentration of 250 µM was added. After mixing for 10 s, the total volume was transferred to a standard 10 x 10 mm quartz glass cuvette sealed with a screw cap. Outside of the glovebox, the cuvette was irradiated with one LED-stick (470 ± 20 nm, 45 ± 5 $mW\cdot cm^{-1}$) in the setup shown in Figure 1 (for room temperature measurements) or Figure 2 (for higher temperatures).

Table S1. Reaction conditions used for thermal and photocatalytic NAD^+ reduction by **Ru(pp)Rh** and **Ru(p(Ph)p)Rh**. All compounds were added under argon atmosphere. The final catalytic solution was prepared in the glovebox (argon) and transferred from GC-vials into standard inert cuvettes.

**Thermal NAD^+ reduction**	**Light-driven Rh(I)-formation[a]**	**Photocatalytic NAD^+ reduction[a]3**
V = 3 mL	V = 3 mL	V = 3 mL
MeCN:H_2O = 1:9	MeCN:H_20 = 1:9	MeCN:H_20 = 1:2
5 µM RuRh-catalyst	10/20 µM RuRh catalyst	5 µM RuRh-catalyst
50 mM $NaHCO_2$	0.12 M TEA	0.12 M TEA
	-	0.10 M NaH_2PO_4
250 µM NAD^+	-	250 µM NAD^+

[a] Catalysis was performed under argon atmosphere.

1.8 Time-dependent Rh(I) formation

Kinetic UV/vis absorbance measurements were performed on an Avantes AvaSpec-ULS2048CL detector unit coupled with an AVA AvaLight-DH-S-BAL light source. Light source, cuvette holder and detector were connected with fibreoptic cables. Measurements were performed in standard 10 x 10 mm cuvettes. Spectra were measured every 2 seconds. Irradiation was performed with one LED-stick (λ_{max} = 470 ± 20 nm, 45 ± 5 mW·cm^{-1}) installed parallel to the cuvette under constant air-cooling by two fans to ensure that the sample was not heated during measurement (see Figure S3).

2. Synthesis

2.1 Synthesis of $[(tbbpy)_2Ru(pp)Rh(Cp^*)Cl]Cl(PF_6)_2$ (Ru(pp)Rh)

To a 10 mL dichloromethane (DCM) solution containing 40 mg (31.1 µmol, 1 eq.) of $[(tbbpy)_2Ru(pp)](PF_6)_2$ a solution of 9.6 mg (15.55 µmol, 0.5 eq.) di-µ-chloro-bis[chloro(pentamethyl-cyclopentadienyl)rhodium(III)], dissolved in 5 mL DCM, was added dropwise within 5 minutes. After stirring for 2 h at room temperature, the solvent was removed in vacuo. At this stage, the red compound was already NMR pure and needed no further purification. Yield: 48.1 mg (97 %, 30.2 µmol) of **Ru(pp)Rh**.

^{1}H NMR (400 MHz, CD_3CN) δ 9.41 – 9.25 (m, $2H_{2'/9'}$), 8.94 – 8.83 (m, 1H), 8.75 – 8.45 (m, 5H), 8.45 – 7.89 (m, 8H), 7.88 – 7.79 (m, 1H), 7.75 – 7.53 (m, 4H), 7.48 (dddd, *J* = 11.0, 6.4, 4.0, 2.1 Hz, 3H), 7.40 – 7.32 (m, 1H), 7.32 – 7.20 (m, 1H), 1.78 (dd, *J* = 10.9, 3.1 Hz, $15H_{Me}$), 1.48 – 1.34 (m, $36H_{tb/tb'}$).

^{13}C NMR (101 MHz, CD_3CN) δ 163.73, 163.59, 158.15, 158.08, 157.88, 153.95, 153.68, 153.58, 153.54, 153.42, 153.35, 153.27, 152.76, 152.72, 152.66, 152.30, 152.22, 152.19, 152.08, 152.03, 149.33, 149.28, 149.22, 149.16, 149.10, 146.80, 146.67, 146.61, 140.46, 140.40, 140.37, 138.63, 138.56, 138.25, 138.20, 137.69, 137.68, 137.61, 137.57, 136.10, 136.01, 135.97, 135.92, 135.68, 135.63, 131.91, 131.84, 131.79, 131.73, 131.55, 131.48, 131.43, 131.35, 131.32, 130.95, 130.93, 130.90, 128.48, 128.06, 127.98, 127.91, 127.64, 127.61, 127.17, 127.13, 127.11, 125.61, 125.49, 122.64, 122.56, 98.54, 98.49, 36.40, 36.39, 36.35, 36.32, 30.54, 30.49, 9.39, 9.35.

ESI-MS: 707.18494 $[M – Cl – PF_6]^{2+}$ (calculated: 707.18445 m/z); 991.24998 $[2 M – 2 Cl – PF_6]^{3+}$ (calculated: 991.23571 m/z).

2.2 Synthesis of $[(tbbpy)_2Ru(p(Ph)p)Rh(Cp^*)Cl]Cl(PF_6)_2$ (Ru(p(Ph)p)Rh)

After dissolving 100 mg (73.4µmol, 1 eq.) of $[(tbbpy)_2Ru(p(Ph)p)](PF_6)_2$ in 10 mL DCM a solution of 22.7 mg (36.7µmol, 0.5 eq.) di-µ-chloro-bis[chloro(pentamethylcyclopentadienyl)-rhodium(III)], dissolved in 10 mL DCM, was added dropwise within 5 minutes. After stirring for 2 h at room temperature the solvent was removed in vacuo. The reddish solid was dissolved in CAM (chloroform:acetone:methanol; v:v:v = 160:190:150 mL) and purified using size exclusion chromatography (SEPHADEX® - column, CAM used as mobile phase). Collection of the first, deep red fraction yielded the pure product **Ru(p(Ph)p)Rh** in almost 93% (114 mg, 68.3 µmol) yield.

^{1}H NMR (400 MHz, CD_3CN) δ 9.40 – 9.31 (m, 2 $H_{2`,9`}$), 8.89 (dd, J = 8.3, 1.3 Hz, 1 $H_{4`/7`}$), 8.82 (d, J = 8.2 Hz, 1 $H_{4`/7`}$), 8.72 (d, J = 8.0 Hz, 1 $H_{2/9}$), 8.69 – 8.58 (m, 5 $H_{2/9,3/3`bpy}$), 8.37 (s, 1 $H_{6`}$), 8.31 (s, 1 H_6), 8.19 (m, 2 $H_{3`,8`}$), 8.12 (m, 2 $H_{4,7}$), 7.88 (s, 4 H_{ph}), 7.85 – 7.77 (m, 2 $H_{3,8}$), 7.73 (m, 2 H_{6bpy}), 7.50 (m, 4 $H_{5/6`bpy}$), 7.26 m, 2 $H_{5`bpy}$), 1.78 (s, 15 H_{Me}), 1.46 (d, J = 2.3 Hz, 18 H_{tb}), 1.38 (s, 18 $H_{tb`}$).

^{13}C NMR (101 MHz, CD_3CN) δ 163.65, 163.51, 158.14, 158.08, 157.87, 153.24, 153.13, 152.28, 152.12, 152.07, 149.27, 148.35, 146.81, 145.95, 140.90, 140.46, 140.16, 138.58, 138.26, 137.54, 136.09, 131.67, 131.40, 131.22, 131.02, 130.80, 129.08, 128.70, 128.32, 128.03, 127.47, 127.12, 125.54, 125.41, 122.89, 122.79, 98.44, 98.36, 49.63, 36.39, 36.30, 30.59, 30.52, 9.39.

ESI-MS: 448.47601 $[M – 2 PF_6 – Cl]^{3+}$ (calculated: 448.47849 m/z), 745.20027 $[M – PF_6 – Cl]^{2+}$ (calculated: 745.20011 m/z).

3. Supplementary Figures

3.1 Reaction setups

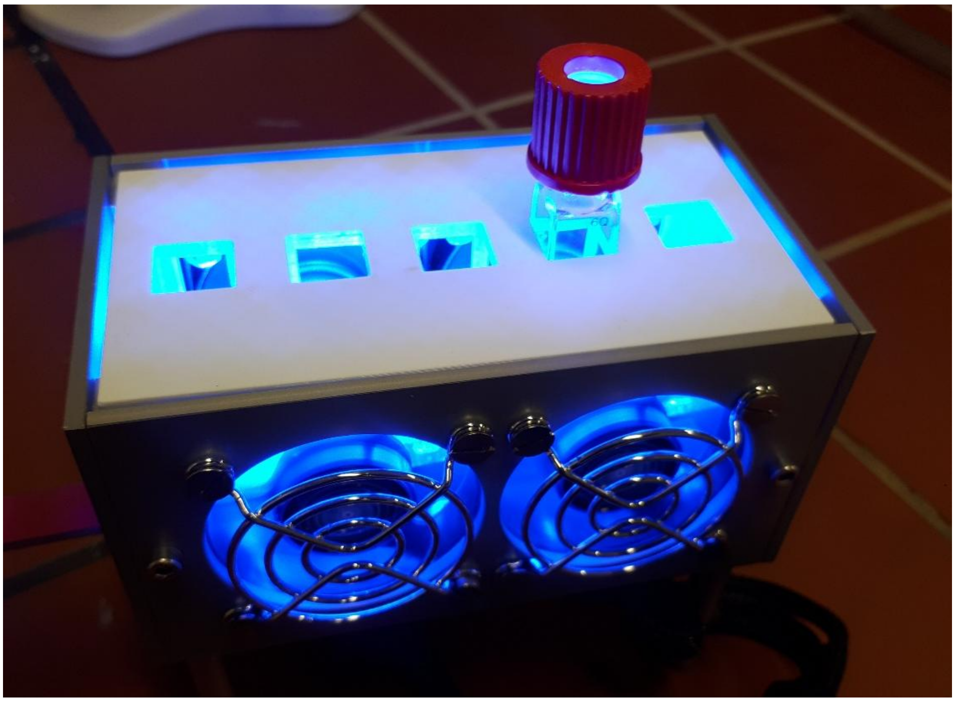

Figure S1. Reaction setup for photostability and photocatalysis measurements of **Ru(pp)Rh** and **Ru(p(Ph)p)Rh** in aerated MeCN at r.t. The 10 x 10 mm standard cuvette was irradiated with one LED-stick (λ_{max} = 470 ± 20 nm, 45 ± 5 $mW\cdot cm^{-1}$) from the bottom.

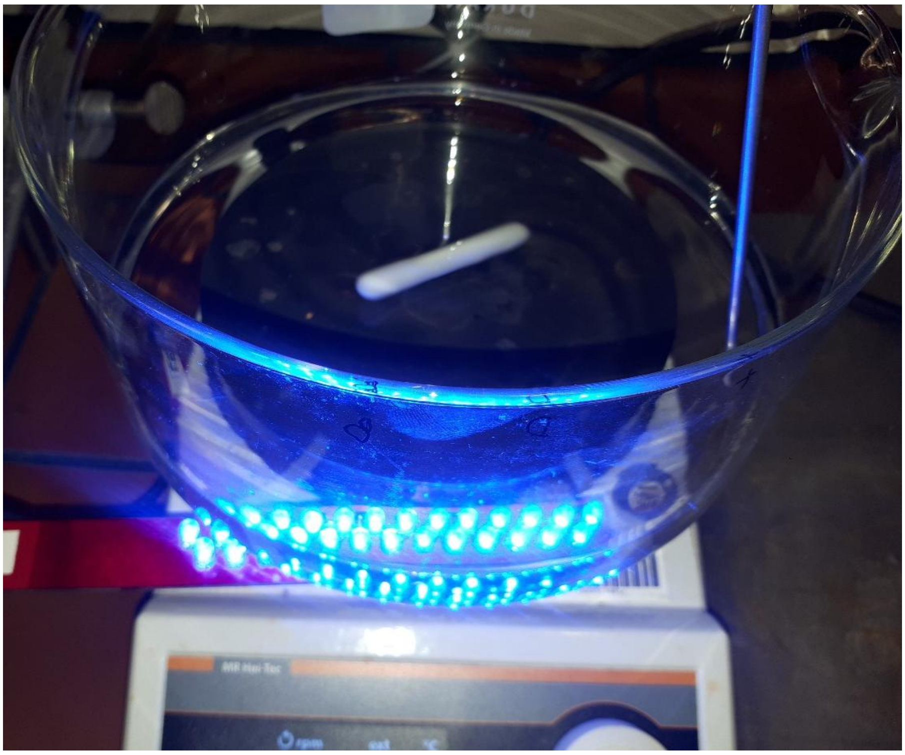

Figure S2. Reaction setup for photocatalytic (with LED stick) and thermal (without LED stick) NAD^+ reduction. The temperature was controlled by utilization of a water bath. The depicted LED-stick emitted at 470 ± 20 nm with an emission power of 45 ± 5 $mW\cdot cm^{-1}$.

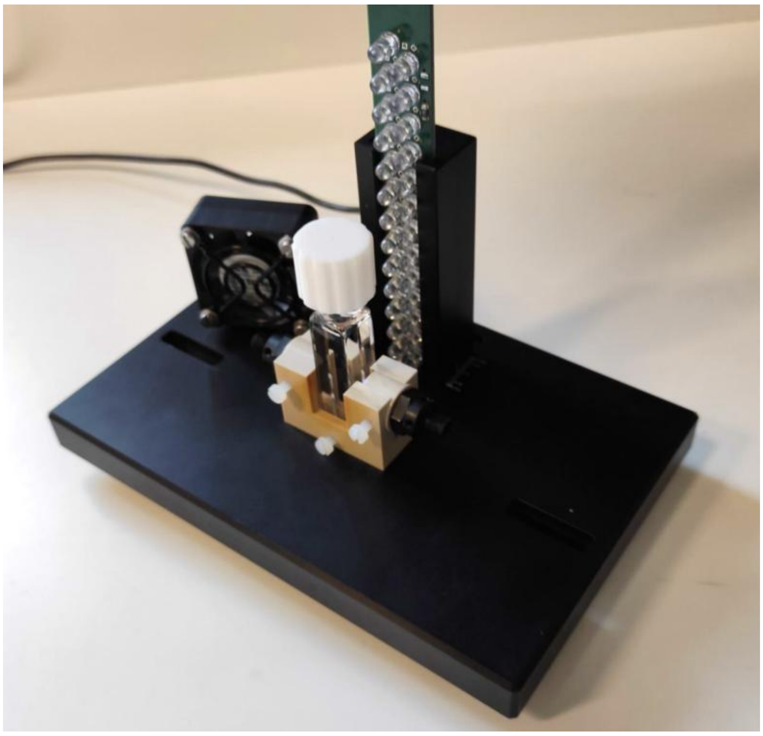

Figure S3. Reaction setup for the determination of the light-driven Rh(III) reduction and subsequent Rh(I) formation.

3.2 NMR experiments

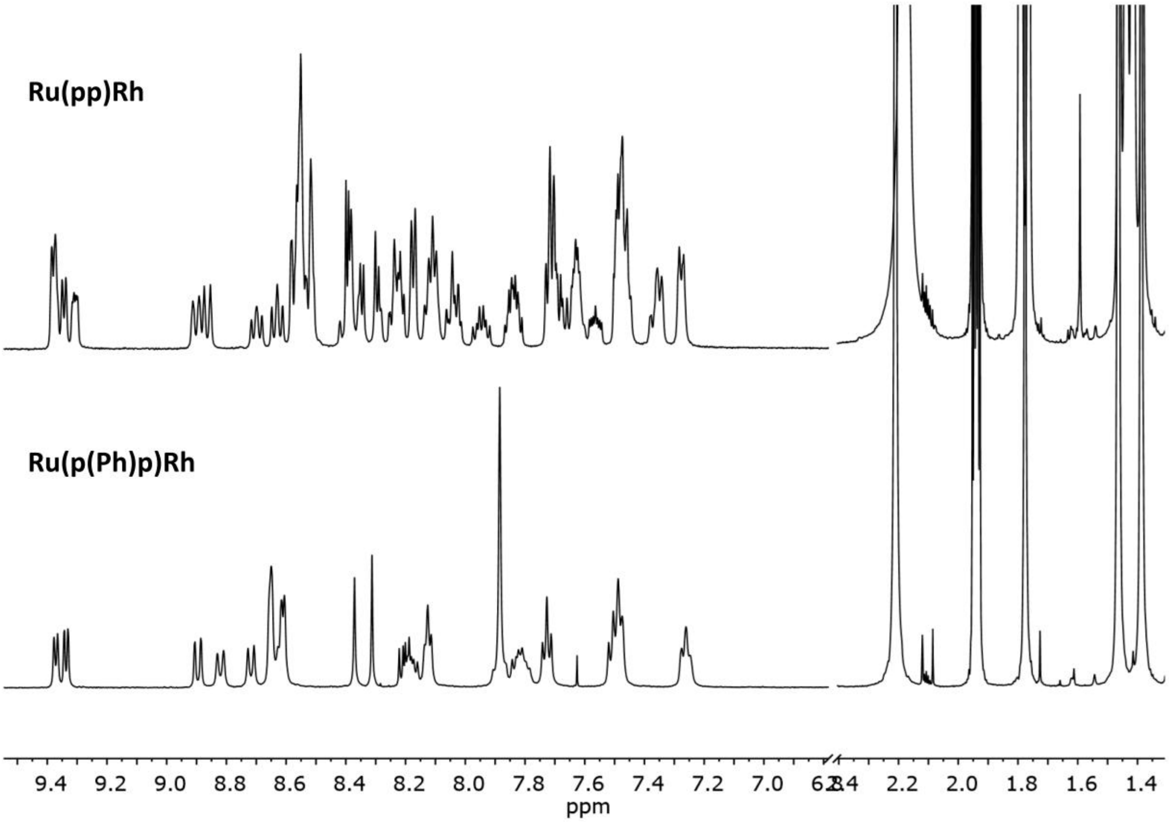

Figure S4. ^{1}H-NMR spectra of **Ru(pp)Rh** (top) and **Ru(p(Ph)p)Rh** (bottom) in MeCN-d_3.

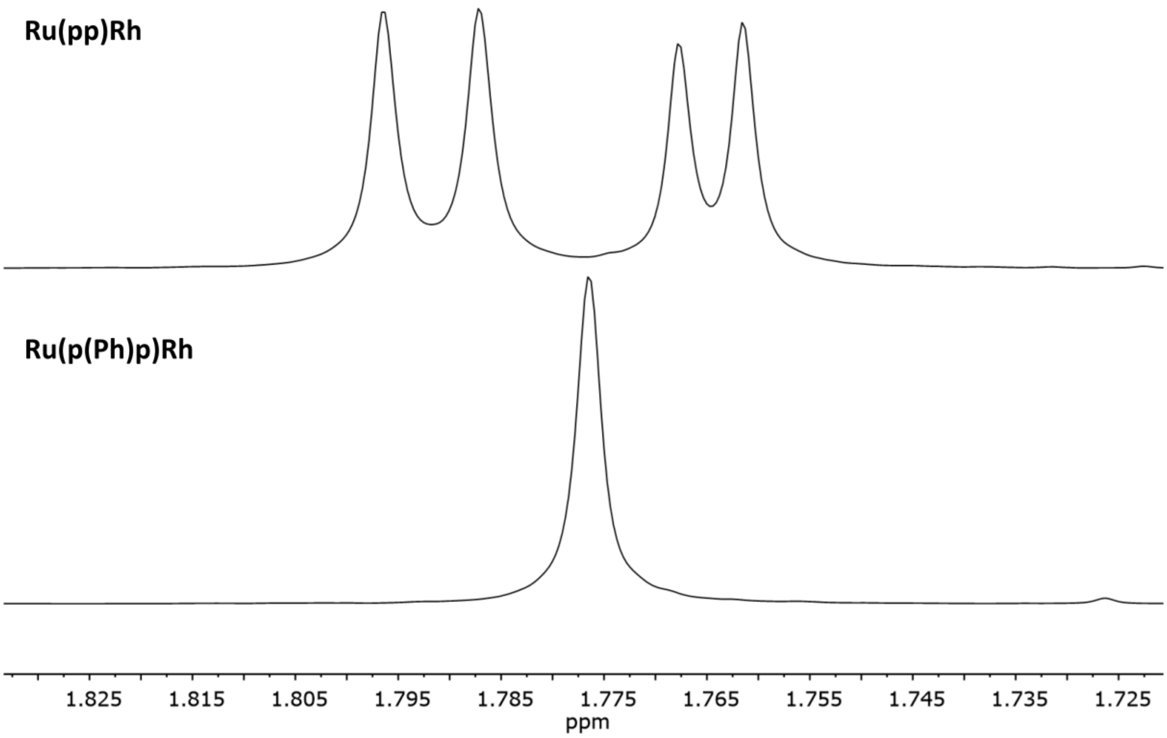

Figure S5. Characteristic ^{1}H-NMR signature of the Cp*-methyl protons of **Ru(pp)Rh** (top) and **Ru(p(Ph)p)Rh** (bottom) in MeCN-d_3.

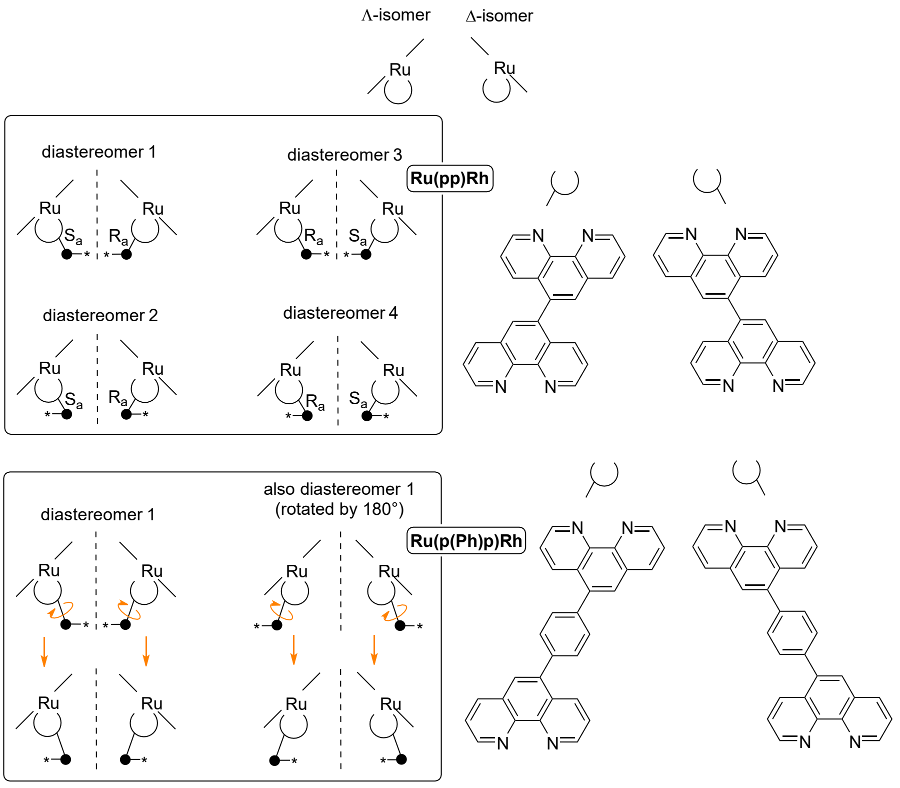

Figure S6: Schematic stereochemical analysis of **Ru(pp)Rh** (top) and **Ru(p(Ph)p)Rh** (bottom) explaining the appearance of different diastereomers that give rise to four different ^{1}H-NMR resonances of the Cp*-associated Me groups in the case of **Ru(pp)Rh** (see Figure S5). The asterisks inside the box indicate the direction in which the Rh-bound Cp* is protruding. The Rh center is indicated by the large black dot. Rotation (indicated by orange color) of the p(Ph)p BL reduced the number of possible diastereomers to only 1; S/R_a indicates left- or right-handed atropisomerism in the case of **Ru(pp)Rh**.

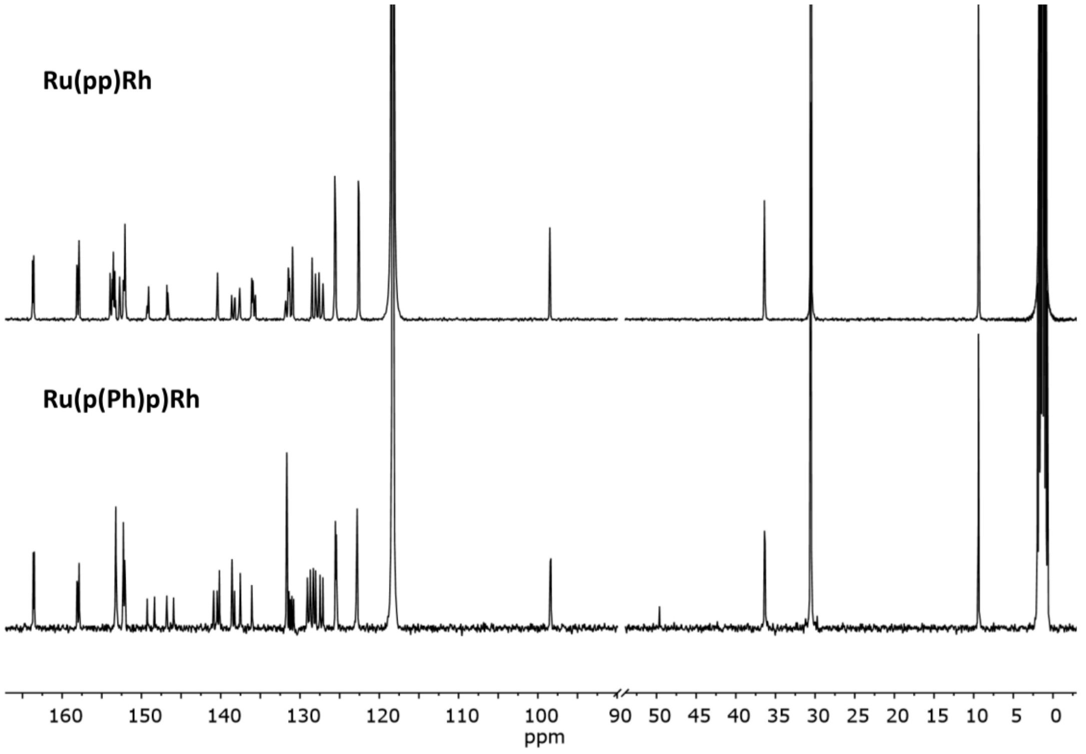

Figure S7. ^{13}C NMR spectra of **Ru(pp)Rh** (top) and **Ru(p(Ph)p)Rh** (bottom) in MeCN-d_3.

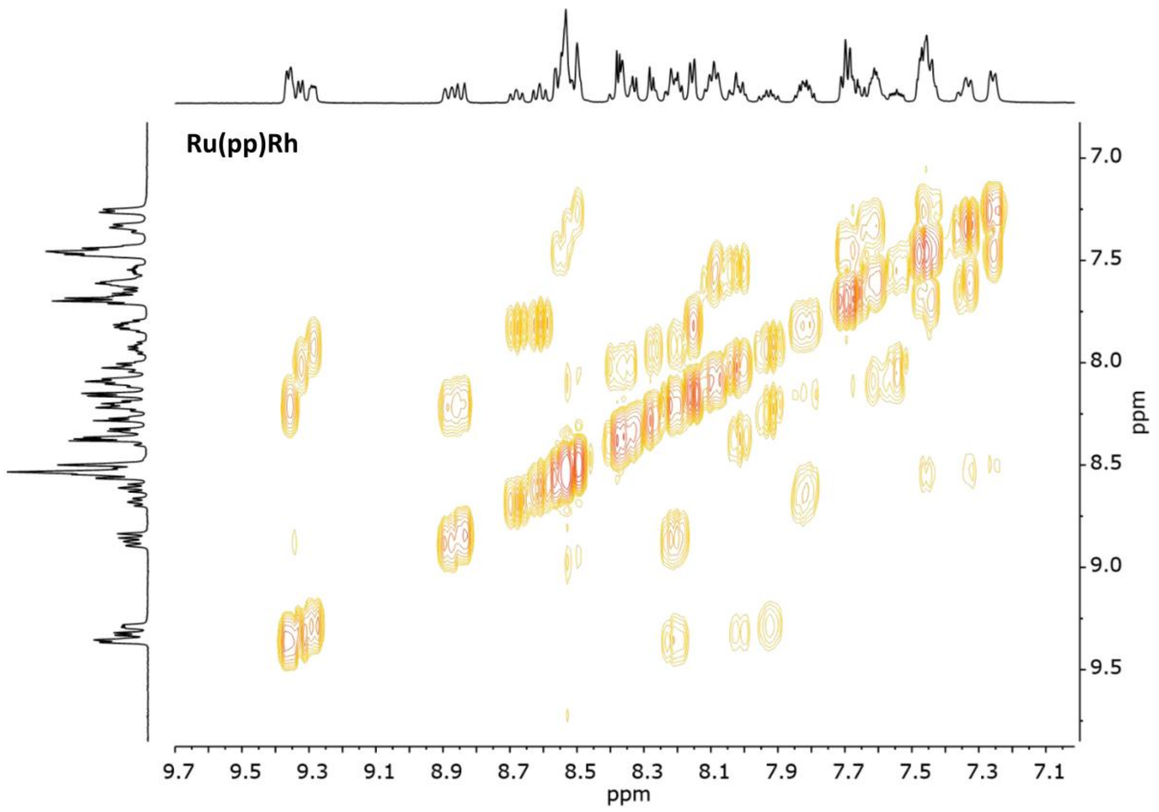

Figure S8. H,H-COSY NMR spectrum of **Ru(pp)Rh** in MeCN-d_3.

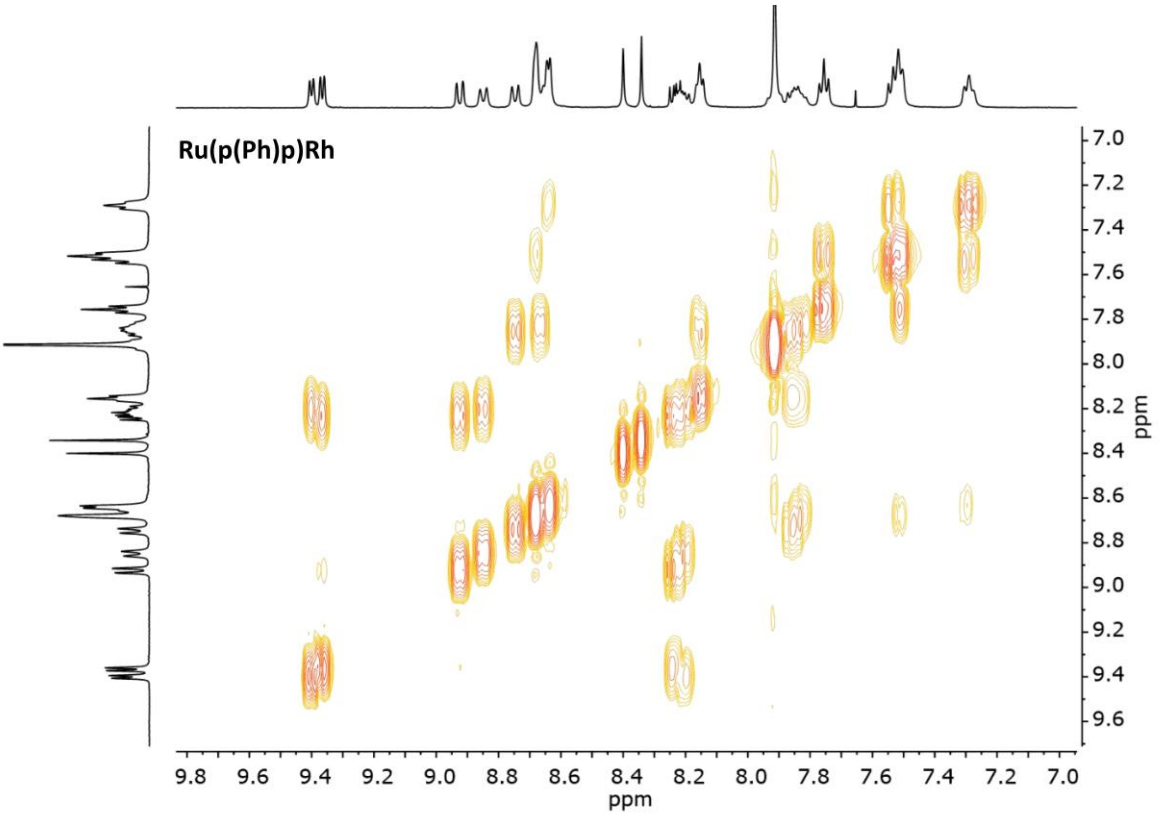

Figure S9. H,H-COSY NMR spectrum of **Ru(p(Ph)p)Rh** in MeCN-d_3.

3.3 Mass spectrometry

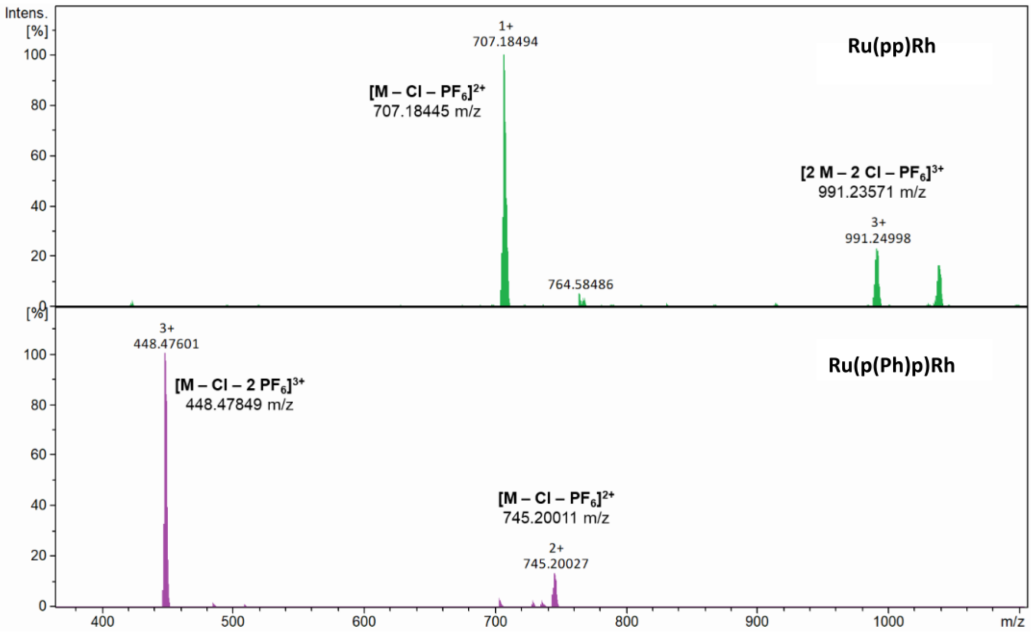

Figure S10. ESI-MS spectra of **Ru(pp)Rh** (top) and **Ru(p(Ph)p)Rh** (bottom) with peak assignment.

3.4 Transient absorption spectroscopy

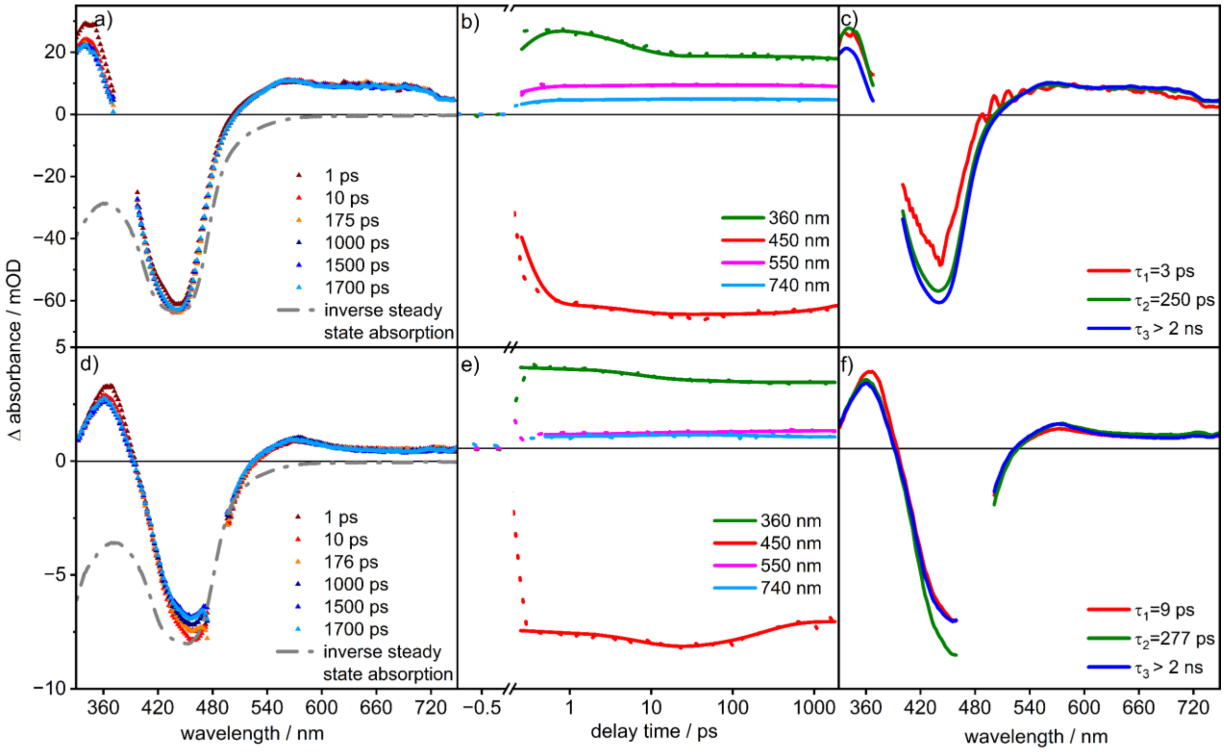

Figure S11. a) and d) show the transient absorption spectrum of **Ru(pp)Rh** under air in MeCN at excitation wavelengths of 400 (a) and 480 nm (d), respectively, in the wavelength range of 340 to 750 nm (0.4 mW). b) and e) show the kinetics with respect to their delay time in ps. c) and f) show the decay associated spectra (DAS) in the wavelength range of 340 to 750 nm.

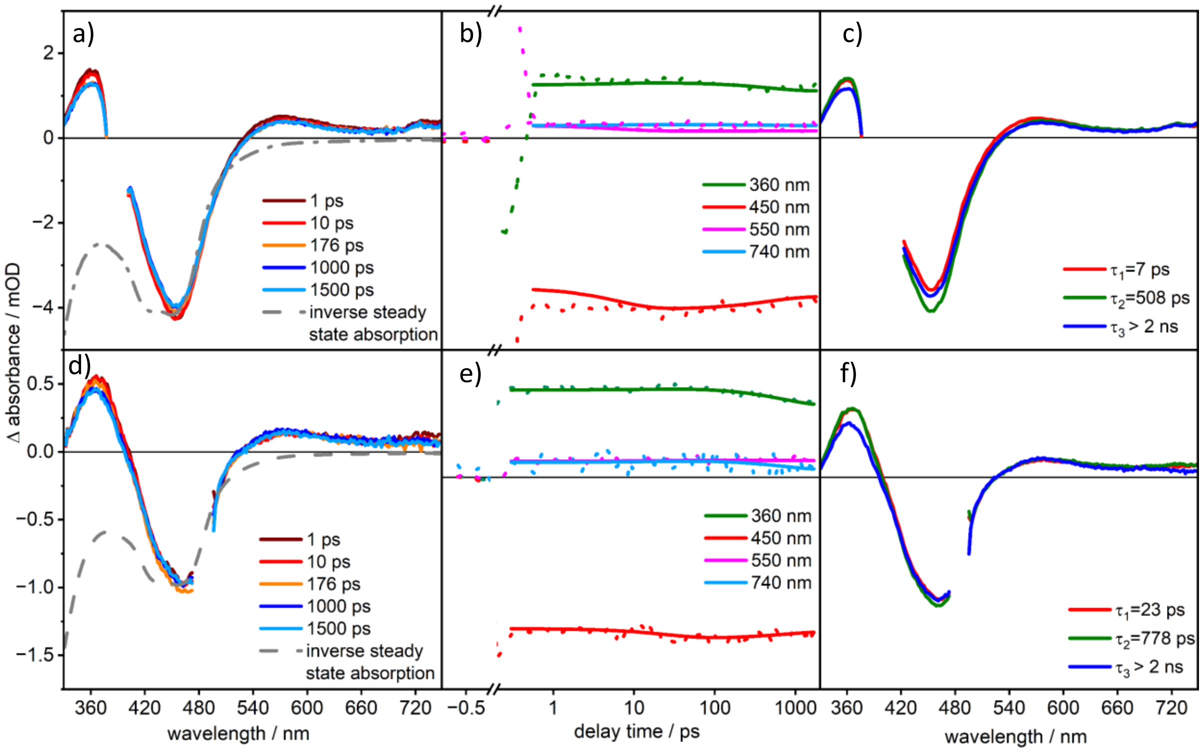

Figure S12. a) and d) show the TA spectra of **Ru(p(Ph)p)Rh** in diethylene glycol (DEG) solvent at excitation wavelengths of 400 and 480 nm, respectively, in the wavelength range of 340 to 750 nm (0.4 μJ/ pulse). b) and e) show the kinetics with respect to their delay time in ps. c) and f) show the decay-associated spectra (DAS) in the wavelength range of 340 to 750 nm.

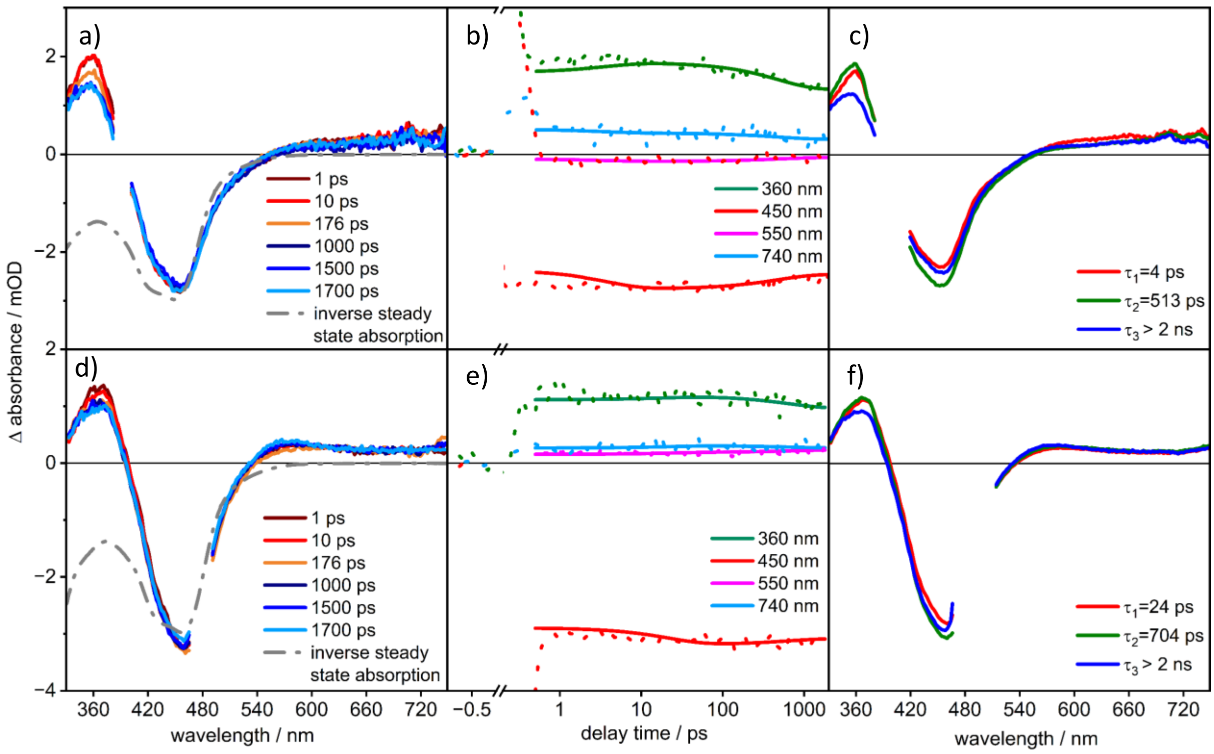

Figure S13. a) and d) show the TA spectra of **Ru(pp)Rh** in DEG at excitation wavelengths of 400 and 480 nm, respectively, in the wavelength range of 340 to 750 nm (0.4 μJ/ pulse). b) and e) show the kinetics with respect to their delay time in ps. c) and f) show the decay-associated spectra (DAS) in the wavelength range of 340 to 750 nm.

3.5 Photostability

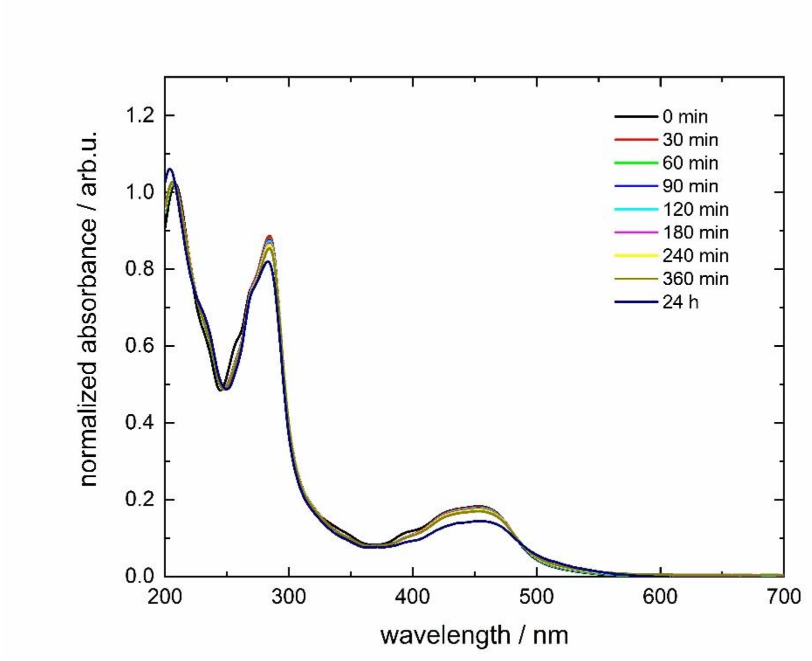

Figure S14. Photostability of **Ru(pp)Rh** in aerated MeCN at r.t. irradiated with one LED-stick (470 ± 20 nm, 45 ± 5 mW·cm^{-1}).

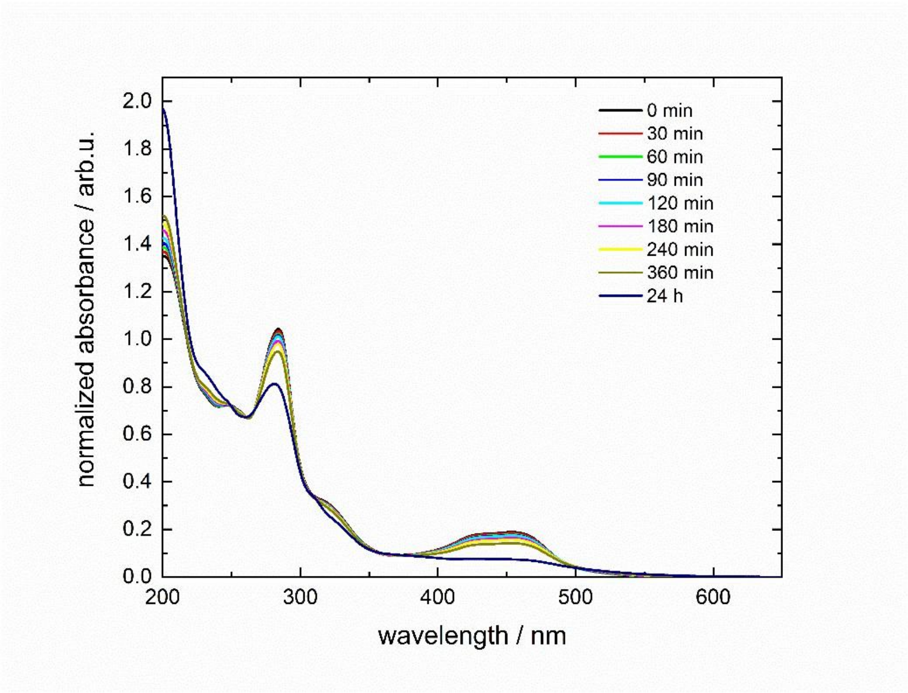

Figure S15. Photostability of **Ru(p(Ph)p)Rh** in aerated MeCN at r.t. irradiated with one LED-stick (470 ± 20 nm, 45 ± 5 mW·cm^{-1}).

Table S2. Photostability data as percentage of **Ru(pp)Rh** and **Ru(p(Ph)p)Rh** compared to the absorption maxima at the beginning after defined time intervals in air-saturated MeCN by irradiation with one LED-stick (470 ± 20 nm, 45 ± 5 mW·cm^{-1}).

time	Ru(pp)Rh[a]	Ru(p(Ph)p)Rh[b]
0 min	0	0
30 min	1.19	2.08
60 min	2.44	5.22
90 min	2.98	6.33
120 min	3.31	7.79
180 min	4.22	13.20
240 min	5.28	16.48
360 min	7.13	24.74
**24 h**	**21.08**	**60.83**

[a] At 451 nm the loss of absorption was highest, while the photolyzed product provided a low absorption.[4]
[b] At 453 nm the loss of absorption was the maximum, while the photolyzed product provided a low absorption.[4]

3.6 Photocatalysis

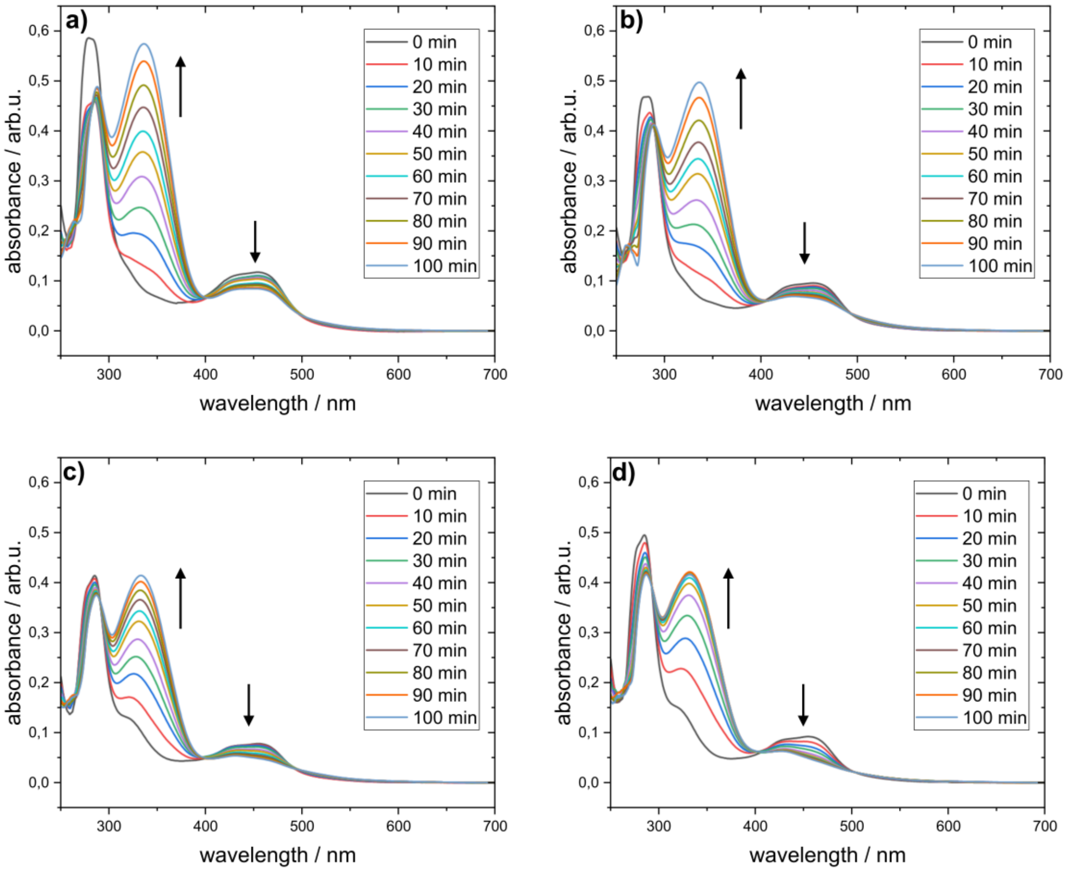

Figure S16. In-situ UV/Vis spectroscopic data of photocatalytic NADH formation by **Ru(pp)Rh** at 25°C (panel a) and 45°C (panel B) or **Ru(p(Ph)p)Rh** at 25°C (panel c) or 45°C (panel d), respectively, in 2:1 (H_2O:MeCN, V_{total} = 3 mL) with 5 µM photocatalyst, 250 µM NAD^+, 0.12 M triethylamine, 0.10 M NaH_2PO_4, irradiated with one LED-stick (470 ± 20 nm, 45 ± 5 mW·cm^{-1}).

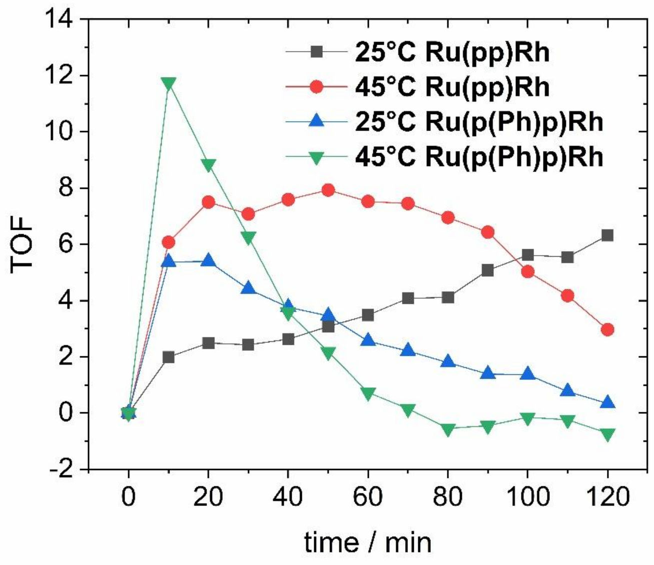

Figure S17. Turnover frequency (TOF) of NAD^+ to NADH reduction for **Ru(pp)Rh** and **Ru(p(Ph)p)Rh** at 25°C and 45°C, respectively in 2:1 (H_2O:MeCN, V_{total} = 3 mL) with 5 µM RuRh photocatalyst, 250 µM NAD^+, 0.12 M triethylamine, 0.10 M NaH_2PO_4, irradiated with one LED-stick (470 ± 20 nm, 45 ± 5 mW·cm^{-1}).

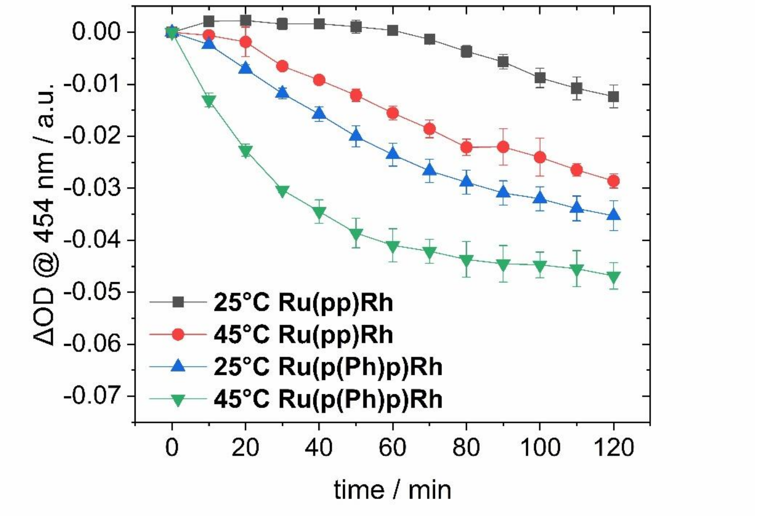

Figure S18. Differential optical density at the maximum MLCT absorption (λ_{max} = 454 nm) of **Ru(pp)Rh** and **Ru(p(Ph)p)Rh** during catalysis after defined time intervals. Conditions: NAD^+ to NADH reduction for **Ru(pp)Rh** and **Ru(p(Ph)p)Rh** at 25°C and 45°C, respectively in 2:1 (H_2O:MeCN, V_{total} = 3 mL) with 5 µM RuRh photocatalyst, 250 µM NAD^+, 0.12 M triethylamine, 0.10 M NaH_2PO_4, irradiated with one LED-stick (470 ± 20 nm, 45 ± 5 mW cm^{-1}).

3.7 Thermal catalysis

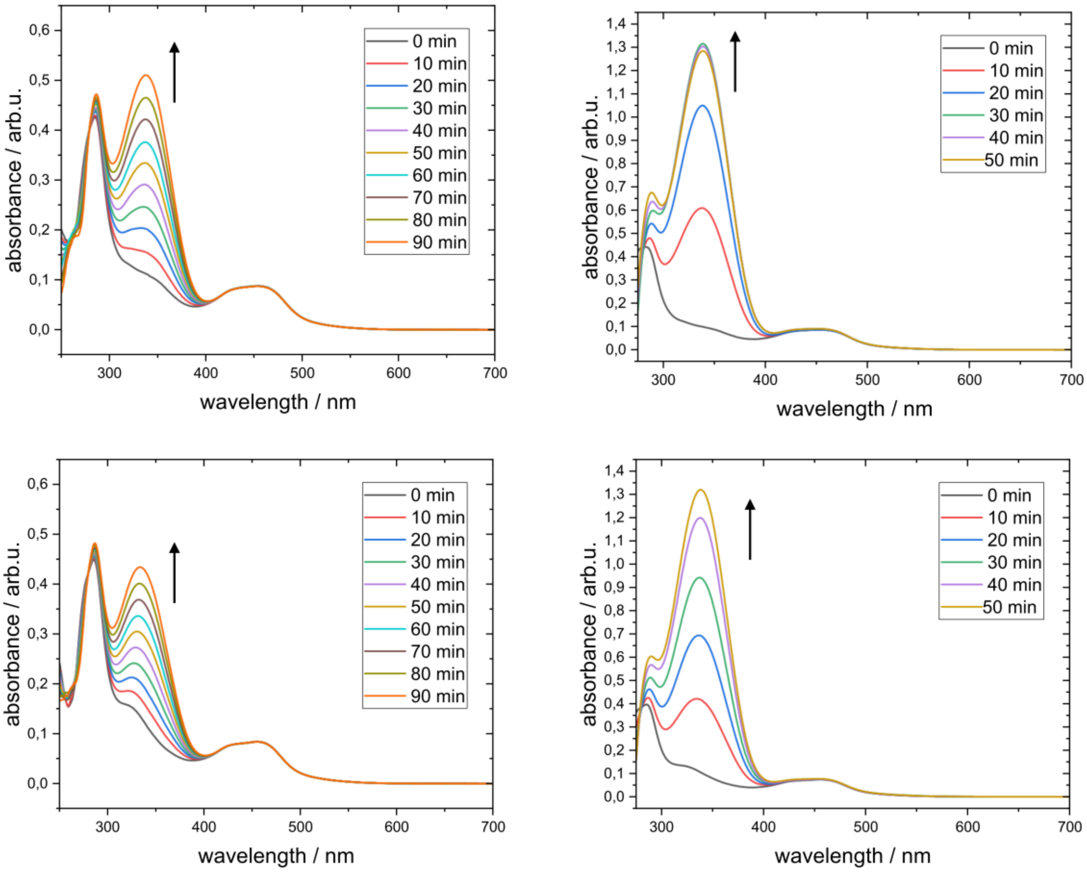

Figure S19. In-situ UV/Vis spectroscopic data of thermal, formate-driven NADH formation by **Ru(pp)Rh** at 25°C (panel a) and 45°C (panel B) or **Ru(p(Ph)p)Rh** at 25°C (panel c) or 45°C (panel d), respectively, in 9:1 = H_2O:MeCN (v:v) containing 50 mM $NaHCO_2$ and 250 µM NAD^+, by 5 µM catalyst,

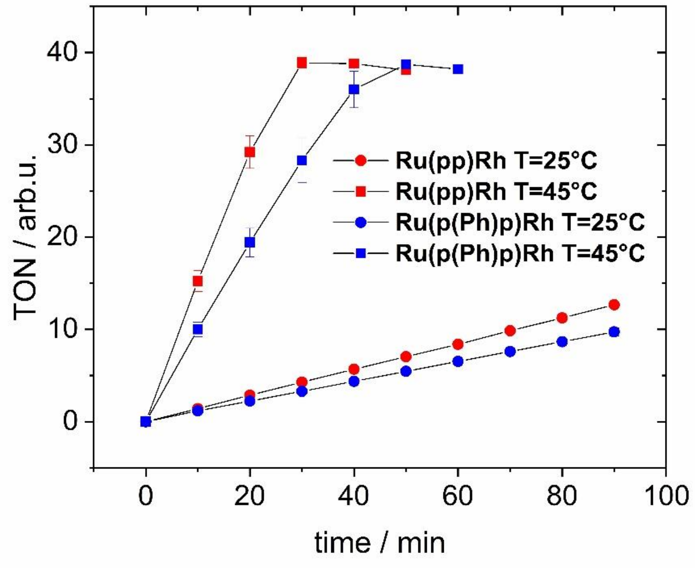

Figure S20. TONs for the formate-driven thermal nicotinamide reduction of **Ru(pp)Rh** and **Ru(p(Ph)p)Rh** at 25°C and 45°C, respectively, by 5 µM catalyst, 50 mM $NaHCO_2$ and 250 µM NAD^+ reaction mixture (9:1 = H_2O:MeCN (v:v)). Catalysis stops due to consumption of the NAD^+ present in solution.

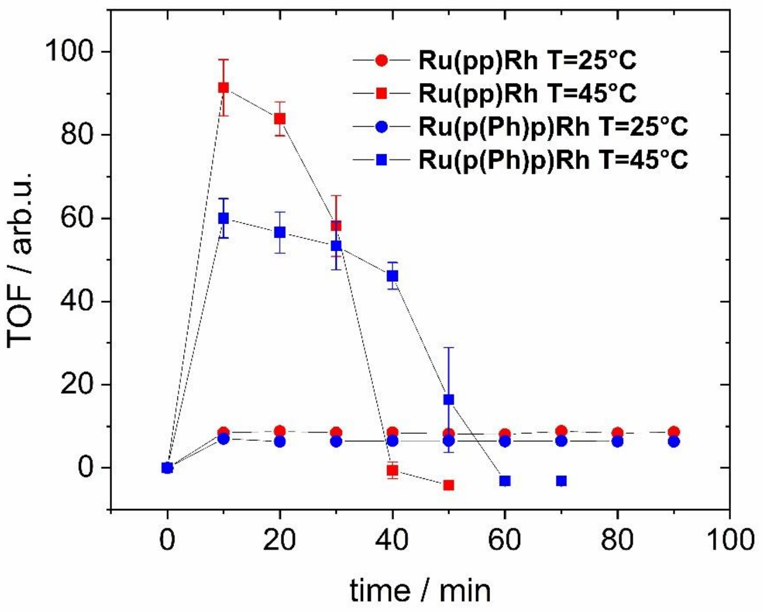

Figure S21. TOFs for the formate-driven thermal nicotinamide reduction of **Ru(pp)Rh** and **Ru(p(Ph)p)Rh** at 25°C and 45°C, respectively, by 5 µM catalyst, 50 mM $NaHCO_2$ and 250 µM NAD^+ reaction mixture (99:1 = H_2O:MeCN (v:v)). Catalysis stops due to consumption of the NAD^+ present in solution.

3.8 Rh(I) formation

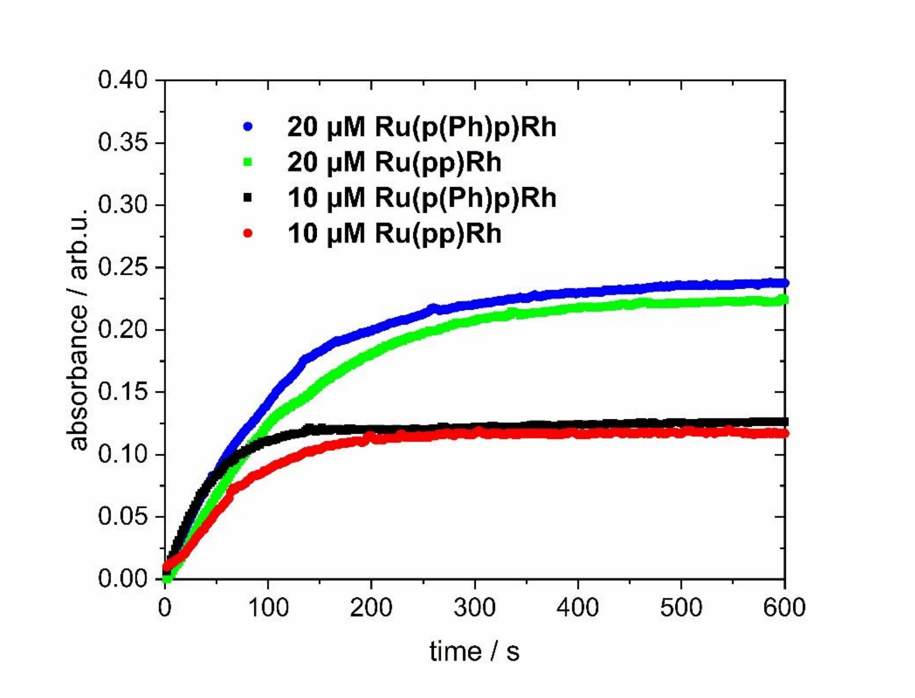

Figure S22. Time-dependent photochemical reduction (one LED-stick, 470 ± 20 nm, 45 ± 5 mW·cm^{-1}) of **Ru(pp)Rh** and **Ru(p(Ph)p)Rh** followed at 680 nm in presence of 0.12 M TEA in 9:1 (v:v) H_2O:MeCN.

3.9 Theoretical Calculations

Table S3. TDDFT calculated excited singlet states obtained from the FC geometry of **Ru(p(Ph)p)Rh**. Details of the lowest 12 excited states are given, alongside key higher energy states. CDDs are given for each transition (red→blue) and the relevant molecular orbitals are shown below in blue and green.

**Ru(p(Ph)p)Rh**	E / eV	λ / nm	*f*	Wgt %	From	To	CDD
S_1	2.44	508	0.001	35<br>30<br>27	272<br>272<br>272	274<br>275<br>276	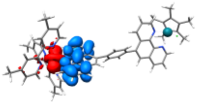
S_2	2.50	496	0.001	11<br>16<br>69	272<br>272<br>272	274<br>275<br>276	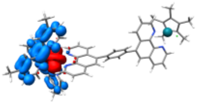
S_3	2.52	492	0.001	66<br>25	272<br>272	277<br>278	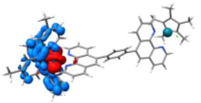
S_4	2.64	469	0.008	29<br>24<br>32	270<br>270<br>270	274<br>275<br>276	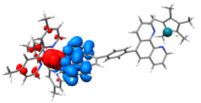
S_5	2.67	465	0.015	15<br>15<br>16<br>30	271<br>271<br>272<br>272	274<br>275<br>275<br>278	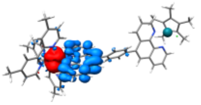
S_6	2.70	460	0.010	13<br>16<br>19<br>29<br>12	270<br>270<br>270<br>271<br>271	274<br>275<br>276<br>277<br>278	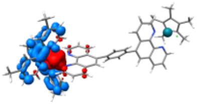
S_7	2.71	458	0.001	17<br>65	269<br>269	273<br>279	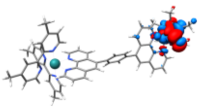
S_8	2.71	458	0.051	25<br>21<br>22<br>10	271<br>271<br>271<br>272	274<br>275<br>276<br>278	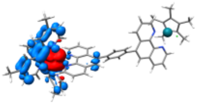
S_9	2.79	445	0.113	39<br>29	270<br>271	277<br>276	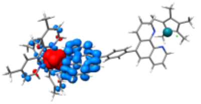
S_{10}	2.83	439	0.164	43<br>31<br>11	270<br>271<br>271	276<br>277<br>278	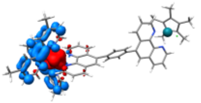
S_{11}	2.89	430	0.135	18<br>48	270<br>270	275<br>278	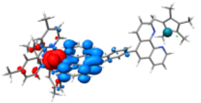
S_{12}	2.91	426	0.026	10<br>29<br>36	271<br>271<br>271	274<br>275<br>278	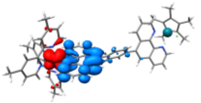
S_{17}	3.09	401	0.030	65	269	280	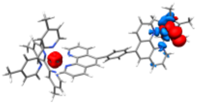

S_{18}	3.15	394	0.015	11<br>14<br>23<br>29	272<br>272<br>272<br>272	274<br>275<br>277<br>278	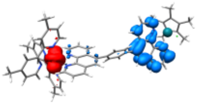
S_{24}	3.42	363	0.163	64	268	273	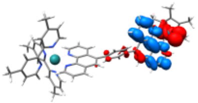
S_{27}	3.55	349	0.156	12<br>24<br>20	268<br>268<br>268	273<br>274<br>275	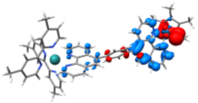
S_{35}	3.66	338	0.242	33<br>22	267<br>268	275<br>275	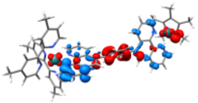

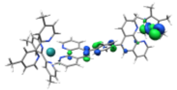
267

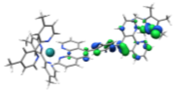
268

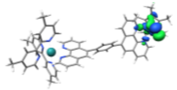
269

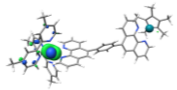
270

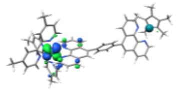
271

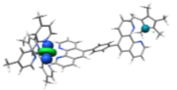
272

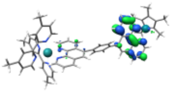
273

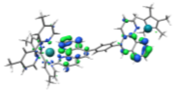
274

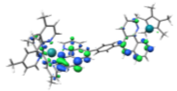
275

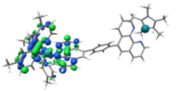
276

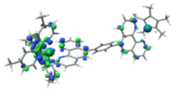
277

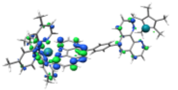
278

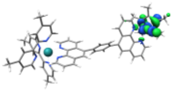
279

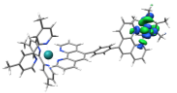
280

Table S4. TDDFT calculated excited singlet states obtained from the FC geometry of **Ru(pp)Rh**. Details of the lowest 12 excited states are given, alongside key higher energy states. CDDs are given for each transition (red→blue) and the relevant molecular orbitals are shown below in blue and green.

**Ru(pp)Rh**	E / eV	λ / nm	*f*	Wgt %	From	To	CDD
S_1	2.42	513	0.001	17<br>52<br>19	252<br>252<br>252	253<br>255<br>256	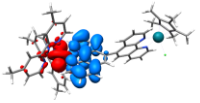
S_2	2.51	494	0.001	22<br>70	252<br>252	255<br>256	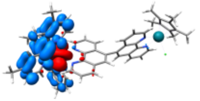
S_3	2.53	491	0.000	65<br>22	252<br>252	257<br>258	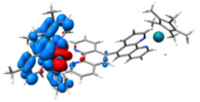
S_4	2.62	473	0.006	25<br>11<br>10<br>24	250<br>250<br>251<br>251	255<br>256<br>253<br>255	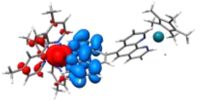
S_5	2.63	471	0.002	26<br>21<br>34	252<br>252<br>252	253<br>254<br>258	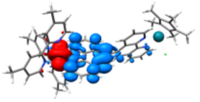
S_6	2.71	458	0.074	13<br>36<br>17	250<br>251<br>251	255<br>255<br>256	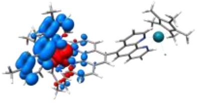
S_7	2.71	458	0.002	10<br>65	249<br>249	253<br>259	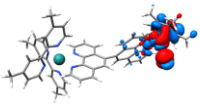
S_8	2.71	457	0.024	24<br>25<br>17	250<br>250<br>251	255<br>256<br>257	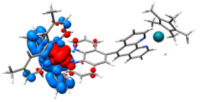
S_9	2.77	448	0.040	10<br>24<br>15<br>22	250<br>250<br>251<br>251	254<br>256<br>253<br>257	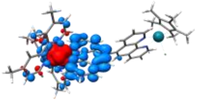
S_{10}	2.83	438	0.163	12<br>23<br>28	250<br>250<br>251	256<br>257<br>256	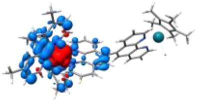
S_{11}	2.85	435	0.158	12<br>20<br>26	250<br>251<br>251	258<br>254<br>258	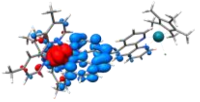
S_{12}	2.87	433	0.0290	24<br>13<br>30<br>10	250<br>250<br>250<br>251	253<br>254<br>258<br>253	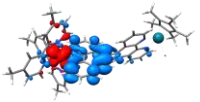
S_{17}	3.08	403	0.016	16<br>12<br>25	249<br>250<br>251	260<br>254<br>254	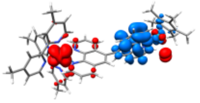

**Ru(pp)Rh**	E / eV	λ / nm	*f*	Wgt %	From	To	CDD
S_{18}	3.09	402	0.015	50	249	260	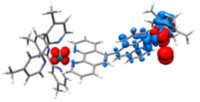
S_{24}	3.41	363	0.107	32	248	253	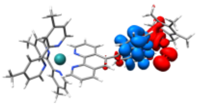
				39	248	254	
				11	248	255	
				12	248	259	
S_{48}	3.90	318	0.098	21	246	253	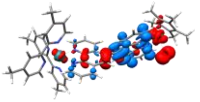
				21	246	254	
				10	248	257	

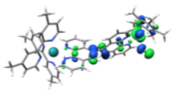
246

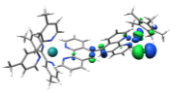
247

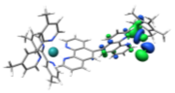
248

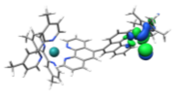
249

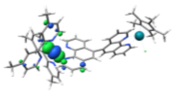
250

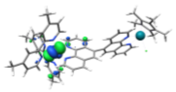
251

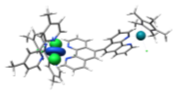
252

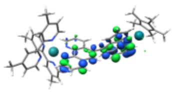
253

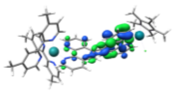
254

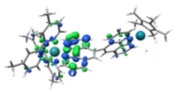
255

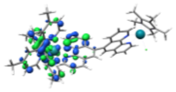
256

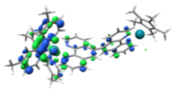
257

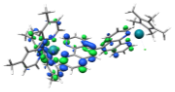
258

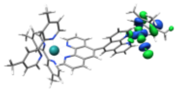
259

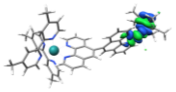
260

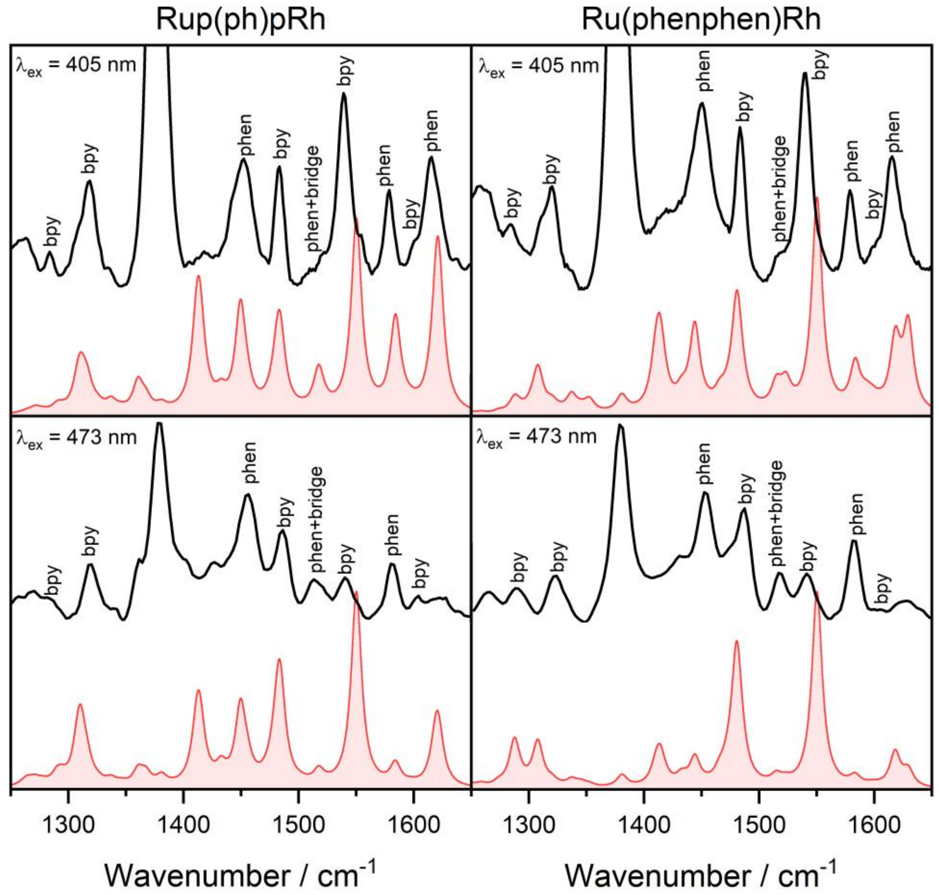

Figure S23. Experimental (black) and simulated (red) resonance Raman (rR) spectra of **Ru(p(Ph)p)Rh** (left) and **Ru(pp)Rh** (right) at excitation wavelengths of 405 (top) and 473 nm (bottom). Vibrational modes have been assigned as reported in the main text. Simulated spectra have been scaled by 0.97, further details regarding the simulated spectra can be found in the computational details.

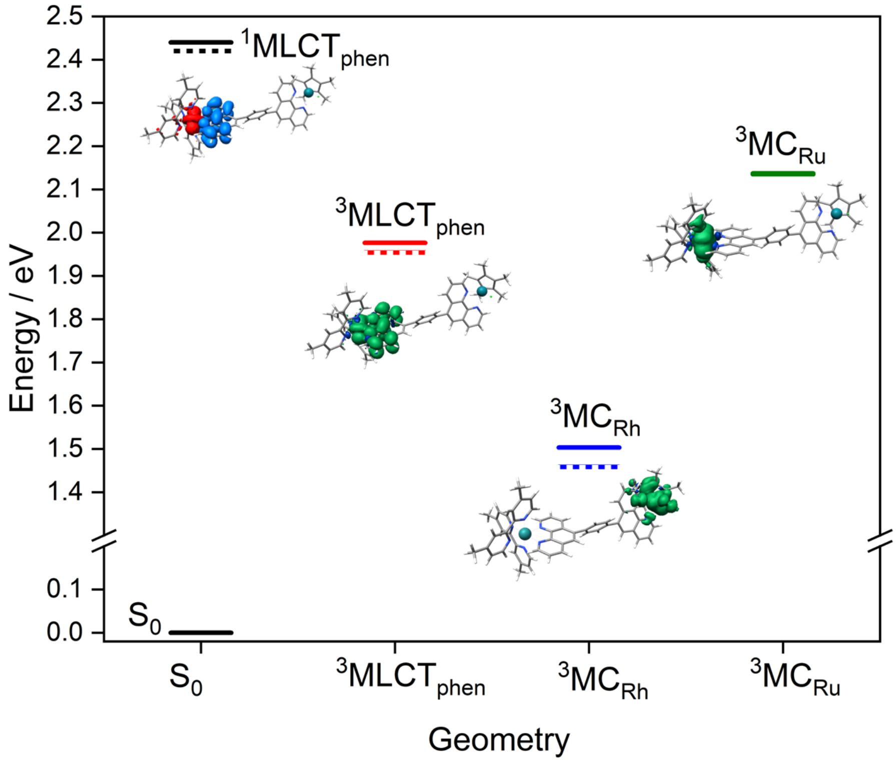

Figure S24. DFT predicted energies of the ^{3}MLCT$_{phen}$, ^{3}MC$_{Rh}$ and ^{3}MC$_{Ru}$ states of **Ru(p(Ph)p)Rh** (solid line) and **Ru(pp)Rh** (dashed line) within their optimized geometries. Where only one line is shown, both complexes possess an isoenergetic excited state. Energies are given relative to the optimised singlet ground state geometry (S_0). The TDDFT predicted energy of the lowest energy ^{1}MLCT$_{phen}$ state (S_1) is included for comparison. Spin density plots of the respective triplet geometries are indicted for **Ru(p(Ph)p)Rh** but are identical for **Ru(pp)Rh**.

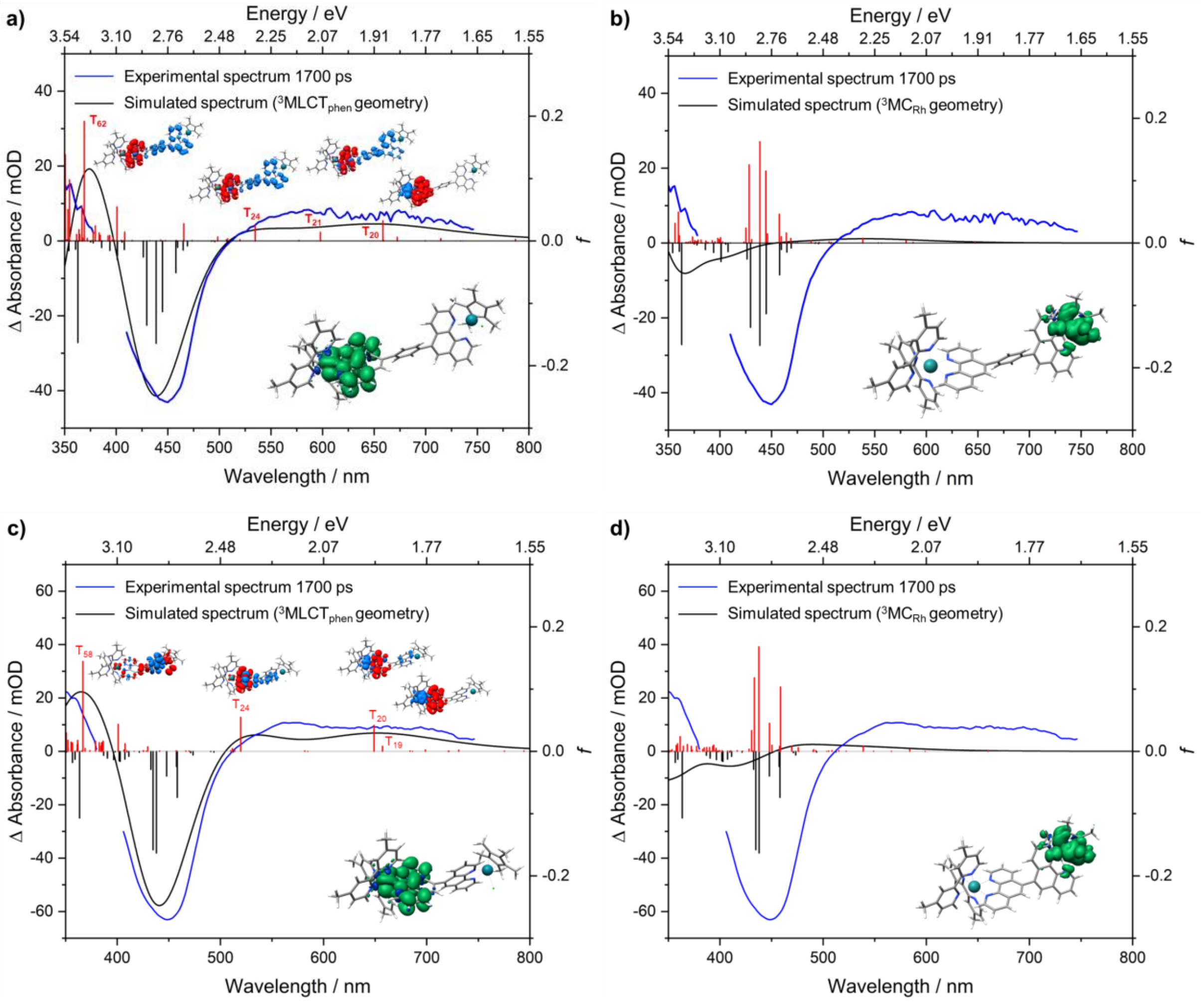

Figure S25. TDDFT simulated transient absorption spectra (black line) compared to experimental transient absorption spectra obtained at 1700 ps (blue line, λ_{ex} = 400 nm) for **Ru(p(Ph)p)Rh** (a, b) and **Ru(pp)Rh** (c, d), from DFT optimised $^{3}MLCT_{phen}$ or $^{3}MC_{Rh}$ geometries. The black bars represent the singlet-to-singlet transitions obtained from the S_0 ground state geometry, while the red bars represent the triplet-to-triplet transitions from the respective $^{3}MLCT_{phen}$ or $^{3}MC_{Rh}$ optimised geometries.

Table S5. Key TDDFT calculated excited triplet states obtained from the optimised $^3MLCT_{phen}$ geometry of **Ru(p(Ph)p)Rh**. CDDs are given for each transition (red→blue).

State	E / eV	λ / nm	*f*	CDD
T_{16}	1.74	715	0.0039	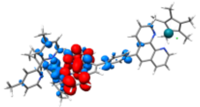
T_{18}	1.84	672	0.0061	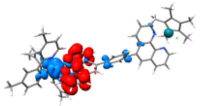
T_{20}	1.88	659	0.0325	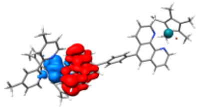
T_{21}	2.07	598	0.0140	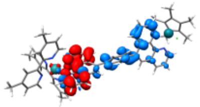
T_{24}	2.32	535	0.0246	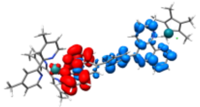
T_{62}	3.36	369	0.1916	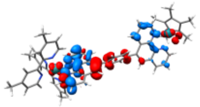

Table S6. Key TDDFT calculated excited triplet states obtained from the optimised $^3MLCT_{phen}$ geometry of **Ru(pp)Rh**. CDDs are given for each transition (red→blue).

State	E / eV	λ /nm	*f*	CDD
T_{19}	1.89	657	0.0081	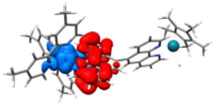
T_{20}	1.91	649	0.0417	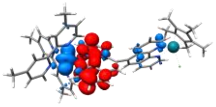
T_{24}	2.39	520	0.0546	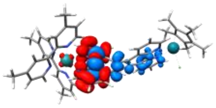
T_{58}	3.38	367	0.1444	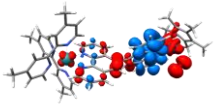

4. References

(1) Brouwer, A. M. Standards for Photoluminescence Quantum Yield Measurements in Solution (IUPAC Technical Report). *Pure Appl. Chem.* **2011**, *83* (12), 2213–2228. https://doi.org/10.1351/PAC-REP-10-09-31.

(2) Suzuki, K.; Kobayashi, A.; Kaneko, S.; Takehira, K.; Yoshihara, T.; Ishida, H.; Shiina, Y.; Oishi, S.; Tobita, S. Reevaluation of Absolute Luminescence Quantum Yields of Standard Solutions Using a Spectrometer with an Integrating Sphere and a Back-Thinned CCD Detector. *Phys. Chem. Chem. Phys.* **2009**, *11* (42), 9850–9860. https://doi.org/10.1039/b912178a.

(3) Zedler, L.; Wintergerst, P.; Mengele, A. K.; Müller, C.; Li, C.; Dietzek-Ivanšić, B.; Rau, S. Outpacing Conventional Nicotinamide Hydrogenation Catalysis by a Strongly Communicating Heterodinuclear Photocatalyst. *Nat. Commun.* **2022**, *13*, 2538. https://doi.org/10.1038/s41467-022-30147-4.

(4) Mengele, A. K.; Müller, C.; Nauroozi, D.; Kupfer, S.; Dietzek, B.; Rau, S. Molecular Scylla and Charybdis: Maneuvering between pH Sensitivity and Excited-State Localization in Ruthenium Bi(Benz)Imidazole Complexes. *Inorg. Chem.* **2020**, *59* (17), 12097–12110. https://doi.org/10.1021/acs.inorgchem.0c01022.
